# Characterizing the regulatory effects of H2A.Z and SWR1-C on gene expression during hydroxyurea exposure in *Saccharomyces cerevisiae*

**DOI:** 10.1371/journal.pgen.1011566

**Published:** 2025-01-21

**Authors:** Hilary T. Brewis, Peter C. Stirling, Michael S. Kobor

**Affiliations:** 1 Department of Medical Genetics, Centre for Molecular Medicine and Therapeutics, BC Children’s Hospital Research Institute, Edwin S.H. Leong Centre for Healthy Aging, University of British Columbia, Vancouver, British Columbia, Canada; 2 Department of Medical Genetics, Terry Fox Laboratory, BC Cancer Research Institute, University of British Columbia, Vancouver, British Columbia, Canada; Institute of Functional Epigenetics, GERMANY

## Abstract

Chromatin structure and DNA accessibility are partly modulated by the incorporation of histone variants. H2A.Z, encoded by the non-essential *HTZ1* gene in *S. cerevisiae*, is an evolutionarily conserved H2A histone variant that is predominantly incorporated at transcription start sites by the SWR1-complex (SWR1-C). While H2A.Z has often been implicated in transcription regulation, *htz1*Δ mutants exhibit minimal changes in gene expression compared to wild-type. However, given that growth defects of *htz1*Δ mutants are alleviated by simultaneous deletion of SWR1-C subunits, previous work examining the role of H2A.Z in gene expression regulation may be confounded by deleterious activity caused by SWR1-C when missing its H2A.Z substrate (apo-SWR1-C). Furthermore, as H2A.Z mutants only display significant growth defects in genotoxic stress conditions, a more substantive role for H2A.Z in gene expression may only be uncovered after exposure to cellular stress. To explore this possibility, we generated mRNA transcript profiles for wild-type, *htz1*Δ, *swr1*Δ, and *htz1*Δ*swr1*Δ mutants before and after exposure to hydroxyurea (HU), which induces DNA replication stress. Our data showed that H2A.Z played a more prominent role in gene activation than repression during HU exposure, and its incorporation was important for proper upregulation of several HU-induced genes. We also observed that apo-SWR1-C contributed to gene expression defects in the *htz1*Δ mutant, particularly for genes involved in phosphate homeostasis regulation. Furthermore, mapping H2A.Z incorporation before and after treatment with HU revealed that decreases in H2A.Z enrichment at transcription start sites was correlated with, but generally not required for, the upregulation of genes during HU exposure. Together this study characterized the regulatory effects of H2A.Z incorporation during the transcriptional response to HU.

## Author summary

Chromatin structure is involved in all aspects of genome function and can be remodelled through a variety of different mechanisms. One foundational aspect of chromatin biology is the incorporation of the histone variant H2A.Z by the SWR1 chromatin remodelling complex (SWR1-C), which results in the formation of structurally distinct regions with diverse biological functions. Despite a general understanding of the basic biology of H2A.Z, many key aspects related to its relationship with SWR1-C, as well as its involvement in gene expression regulation, have remained long-standing enigmas within the field. Here, we take advantage of budding yeast as a model system to examine how preventing H2A.Z incorporation affects the transcriptional response to hydroxyurea (HU) exposure, a genotoxic agent which induces DNA replication stress. In general, we found that H2A.Z played a more prominent role in gene activation than repression during HU exposure, and that its incorporation was essential for many HU-induced genes to achieve wild-type levels of expression. Our results indicate that for future studies to effectively examine the role of H2A.Z in transcription regulation, it is important to account for both the confounding effects of SWR1-C and to examine gene expression before and after gene induction.

## Introduction

Incorporation of histone variants is one of the principal mechanisms that cells use to create structurally and functionally distinct regions of chromatin. H2A.Z is an evolutionarily conserved histone variant in the H2A histone family [1]. Unlike H2A, which is produced in equal amounts to other core histones during S-phase, H2A.Z is encoded by replication-independent genes allowing for variant specific expression and deposition throughout the cell cycle [2]. Essential in higher eukaryotes, H2A.Z has been implicated in a large variety of biological functions including nucleosome turnover, maintenance of heterochromatin and euchromatin boundaries, DNA repair, resistance to genotoxic stress, and transcription regulation [1,3]. It is therefore not surprising that disruption of proper H2A.Z incorporation has been widely connected to human health and disease including memory formation [4–8], development and progression of various cancers [9–14], and phenotypes of the rare genetic developmental disorder Floating Harbor Syndrome [15].

H2A.Z, encoded by the non-essential *HTZ1* gene in *Saccharomyces cerevisiae*, is incorporated into chromatin by the SWR1 complex (SWR1-C), a highly conserved ATP-dependent chromatin-remodeler [16–19]. Replacing H2A in 5–10% of nucleosomes, H2A.Z is primarily incorporated into the +1 nucleosome of the transcription start site (TSS) of approximately 63% of all genes in yeast [20–23]. Despite having very similar three-dimensional structures [24], *in vitro* and *in vivo* experiments provide strong evidence that H2A.Z containing nucleosomes are less stably bound in chromatin than H2A containing nucleosomes [25–30]. However, it is now understood that the impact of H2A.Z on chromatin dynamics is highly contingent on the organism and the nucleosomal context. For example, H2A.Z heterotypic nucleosomes (H2A-H2A.Z-containing) are more stable than H2A.Z homotypic nucleosomes (H2A.Z-H2A.Z-containing) [28,31], and the presence of other histone variants or histone post-translational modifications can further modify histone-histone and histone-DNA interactions [32–34]. In addition to affecting nucleosome stability, H2A.Z occupancy in mammalian models correlates with various transcription-related histone post-translational modifications, such as H3K4 methylation, further indicating a role for H2A.Z in gene expression regulation [3].

While these previous findings suggest a strong relationship between H2A.Z function and gene expression, the role of H2A.Z in transcription regulation has remained a long-standing enigma within the field, particularly in *S. cerevisiae*. One compelling model suggests that H2A.Z occupancy promotes gene activation by facilitating the disassembly of the +1 nucleosome, thereby promoting RNA polymerase II (RNAPII) initiation and elongation [35]. However, microarray experiments in budding yeast have challenged this notion, finding that less than 5% of genes are differentially expressed in the *htz1*Δ mutant in steady-state conditions despite H2A.Z being enriched at the majority of gene promoters [18,22,36–38]. Additionally, previous studies have found no broad correlation between H2A.Z occupancy and gene expression levels, with many H2A.Z-dependent genes exhibiting relatively limited H2A.Z enrichment at their promoter [18,22,23]. However, there is strong evidence to suggest that H2A.Z primarily regulates genes expression during the transcriptional response to cellular stress and changes in environment [3,39]. For example, the *htz1*Δ mutant has defective activation of several inducible genes such as *GAL1*, *PHO5*, and *IMD2* [40–42] and H2A.Z mutants only display significant growth defects when exposed to stress conditions [1]. This then raises the intriguing possibility that the relationship between H2A.Z incorporation and gene activation in *S. cerevisiae* may only be fully elucidated after a change in environment or the introduction of a cellular stress.

H2A.Z mutants are sensitive to several genotoxic agents including hydroxyurea (HU) [42,43], a non-alkylating antineoplastic commonly used to induce DNA replication stress. By increasing the cellular abundance of reactive oxygen species (ROS) and disrupting the production of deoxyribonucleotides (dNTPs), HU activity ultimately leads to cell cycle arrest and DNA damage checkpoint activation [44–46]. Given that the onset of replication stress results in dynamic and substantial changes to the transcriptome [47] and that H2A.Z incorporation is required for proper cell cycle progression (Dhillon *et al.*, 2006; Long *et al.*, 2019), we hypothesized that HU exposure would exacerbate gene expression differences in *htz1*Δ mutants and could clarify the relationship between H2A.Z enrichment and gene expression [48,49]. HU exposure also provides the unique opportunity to examine the curious relationship between SWR1-C and H2A.Z. SWR1-C is required for the incorporation of H2A.Z into chromatin, but deletion of its catalytic subunit, Swr1, produces less severe growth defects during cellular stress compared to *htz1*Δ mutants [16–18]. Furthermore, *htz1*Δ*swr1*Δ mutants have similar growth phenotypes to *swr1*Δ mutants, suggesting that the larger growth defects of *htz1*Δ mutants can be attributed to deleterious activity of SWR1-C missing its H2A.Z substrate (apo-SWR1-C) [42]. Given the growth phenotypes associated with this phenomenon are highly pronounced in HU [42], HU exposure could also serve as a valuable means to determine if the *htz1*Δ mutant’s gene expression patterns are influenced by the presence of apo-SWR1-C.

In an effort to uncover the role of H2A.Z in gene expression regulation in *S. cerevisiae*, we examined the mRNA transcript profiles of *htz1*Δ, *swr1*Δ, and *htz1*Δ*swr1*Δ mutants in both untreated and HU-treated conditions. Analysis of six biological replicates revealed that H2A.Z played a more prominent role in gene activation than gene repression during HU exposure, and that H2A.Z incorporation was important for wild-type expression of several HU-induced genes. We also observed that both the *swr1*Δ and *htz1*Δ*swr1*Δ mutants had less severe gene expression defects compared to the *htz1*Δ mutant, particularly for genes involved in the regulation of phosphate homeostasis. Furthermore, by mapping H2A.Z incorporation at a single-nucleosome resolution before and after HU exposure, we found that decreases in H2A.Z enrichment at transcription start sites correlated with, but was generally not required for, increased mRNA expression of HU-activated genes. Taken together these results indicate that to effectively examine the role of H2A.Z in transcription regulation, it is important to account for the confounding effects of apo-SWR1-C and advisable to examine gene expression before and after gene induction.

## Results

### Apo-SWR1-C contributed to differences in gene expression profiles between wild-type and the *htz1*Δ mutant

**Fig 1:**
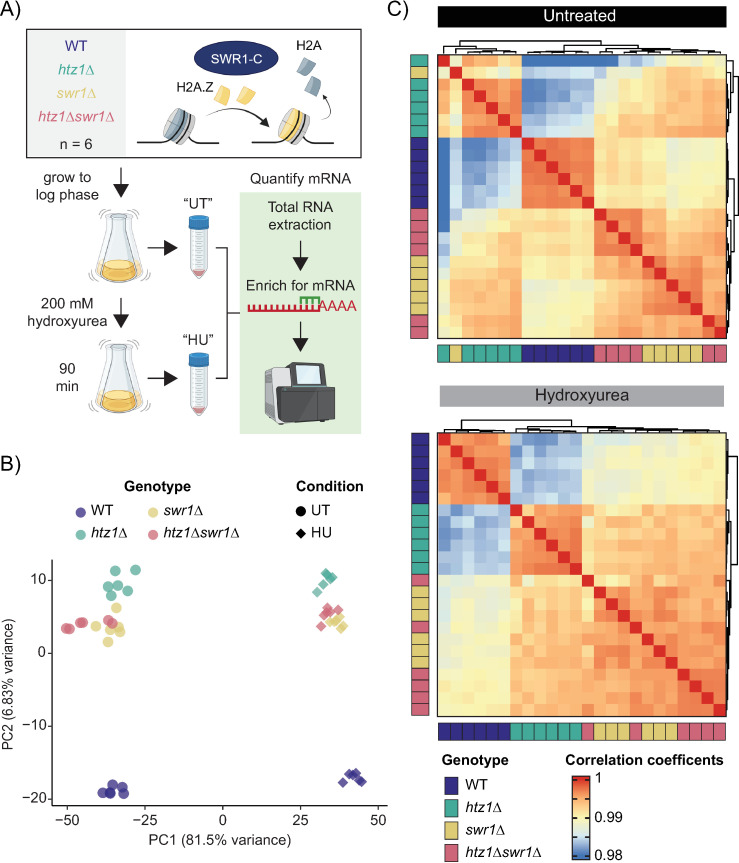
The *htz1*Δ, *swr1*Δ, and *htz1*Δ*swr1*Δ mutants mRNA expression profiles clustered separately from wild-type. (A) Experimental overview of the HU-treatment and RNA-sequencing pipeline. Six biological replicates of wild-type, *htz1*Δ, *swr1*Δ, and *htz1*Δ*swr1*Δ cells were grown to log phase and then split into untreated (UT) and HU-treated (HU) conditions. After extracting total RNA from each sample, mRNA transcripts were enriched and then sequenced using a NextSeq500 illumina platform. Graphic created with BioRender.com. (B) Biplot of PC1 and PC2 generated from a Principal Component Analysis (PCA) of normalized read counts. Each data point represents a single biological replicate (n = 6). (C) Spearman’s correlation coefficients matrix of mRNA expression profiles showed that the *htz1*Δ, *swr1*Δ, and *htz1*Δ*swr1*Δ mutants clustered separately from wild-type in both the untreated and HU-treated conditions. Each coloured cell represents the correlation coefficient between the indicated genotypes for a single biological replicate.

In order to assess the impact H2A.Z incorporation has on gene expression in *S. cerevisiae* during HU exposure, we pursued three primary objectives: 1) characterizing how wild-type gene expression changes in response to HU exposure, 2) determining the influence of H2A.Z incorporation and the presence of apo-SWR1-C on gene expression profiles in both untreated and HU-treated conditions, and finally 3) comparing the patterns of HU-induced gene activation and repression between wild-type and mutants lacking H2A.Z incorporation. To achieve these objectives we sequenced poly(A)-enriched libraries from six biological replicates of wild-type, *htz1*Δ, *swr1*Δ, and *htz1*Δ*swr1*Δ mutants before and after a 90 minute treatment with HU (200 mM) (Fig 1A). The majority of the resulting raw reads were very high in quality (98.4% > Q30). On average, 87% of the cleaned reads were successfully mapped to the reference genome (SacCer3), 79% of which were concordantly aligned and had unique mapping coordinates. As such, every sample had ample genomic coverage (>85X) with between 14.1 – 17.6 million reads available for use in downstream analysis (S1A Fig). After filtering out blacklisted genes (S1A Fig), we obtained Transcripts per Million (TPM) normalized read counts for a total of 5560 genes. *HTZ1* and *SWR1* transcript levels were used to confirm that each sample matched with its expected genotype (S1B Fig).

To first gain an understanding of the overall structure of the dataset we performed both a Principal Component Analysis (PCA) and examined the Spearman’s correlation coefficients comparing the genotypes of the six biological replicates in the untreated and hydroxyurea conditions (Fig 1B and 1C). As expected, the majority of the variance present within the dataset could be explained by the condition (PC1: 81.5%), with a smaller percentage attributed to the genotype of the strains (PC2: 6.83%). Notably, in both untreated and HU-treated conditions, the *htz1*Δ, *swr1*Δ, and *htz1*Δ*swr1*Δ mutants clustered distinctly from wild-type samples. The *swr1*Δ and *htz1*Δ*swr1*Δ mutants, which were nearly indistinguishable from each other, deviated less from wild-type than the *htz1*Δ mutant. Curiously, while the biological replicates of the mutants generally clustered together, the variance (σ^2^) in expression between replicates was significantly higher for all three mutants compared to wild-type, particularly in the untreated condition (S2A Fig). This overall data structure closely resembles the slow growth phenotypes of the *htz1*Δ, *swr1*Δ, and *htz1*Δ*swr1*Δ mutants (S2B Fig), suggesting that shifts in the proportion of cells engaged in each stage of the cell cycle could be driving the distinct gene expression profiles of each genotype [50]. However, this appears to be unlikely as flow cytometry analysis of cellular DNA content revealed no discernable differences in the cell cycle profiles of wild-type cells and the *htz1*Δ, *swr1*Δ, and *htz1*Δ*swr1*Δ mutants in either untreated or HU-treated conditions (S2C Fig). Altogether these results suggested that, while minimal, there are robust observable differences in gene expression profiles between the *htz1*Δ mutant and wild-type that can be partially explained by the deleterious effects of apo-SWR1-C.

### HU exposure activated the environmental stress response and the iron regulon

**Fig 2:**
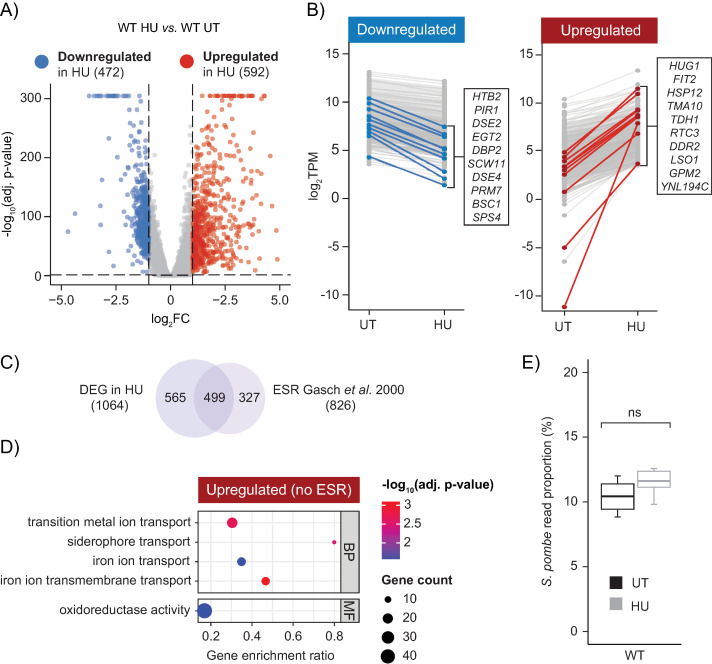
Hydroxyurea exposure activated the Environmental Stress Response and the iron regulon. (A) Volcano plot of DESeq2 results comparing wild-type mRNA expression in untreated and HU-treated conditions. Coloured points are genes with an BH-FDR adjusted p-value < 0.05 and a log_2_FC ≥ 1 (red) or ≤ −1 (blue). (B) Paired dot plot of the downregulated (blue) and upregulated (red) genes found in wild-type after HU exposure. The top 10 genes with the largest log_2_FC in each category are listed. (C) HU-dependent DEGs overlapped with genes in the ESR [52]. (D) Gene Ontology term enrichment analyses of the upregulated DEG genes in HU (ESR genes removed from both submitted and background gene lists). BP = Biological Process, MF = Molecular function. (E) There was no significant difference in the percentage of *S. pombe* reads between the untreated and HU-treated mRNA libraries.

To characterize the impact of HU exposure on gene expression, we employed DESeq2 [51] to identify differentially expressed genes (DEGs) between the wild-type untreated and HU-treated conditions (S3A–S3C Fig). Of the 5560 genes considered in this study, 1064 genes were differentially expressed between the wild-type untreated and HU-treated conditions, with 472 genes downregulated and 592 genes activated after HU induction (Fig 2A and 2B). Nearly half, 499 of 1064, of these DEGs were part of the Environmental Stress Response (ESR), a general stress response pathway in yeast which is activated regardless of the source of stress (Fig 2C) [52]. Gene Ontology term enrichment analysis of the upregulated HU-induced genes that were not part of the ESR, revealed an enrichment of terms associated with the iron regulon, a group of around 30 genes that are required to maintain iron (Fe+) homeostasis within the cell (Figs 2D and S4) [53].

While previously work has also found that HU exposure leads to activation of the ESR and upregulation of the iron regulon in *S. cerevisiae* [47], it is important to note that differential expression analysis can be confounded by differences in mRNA abundance caused by different experimental conditions [54]. To determine if there were significant differences in total RNA abundance between the wild-type untreated and HU-treated conditions we compared the proportion of *S. pombe* spiked-in reads present in their mRNA libraries. We found that both conditions had comparable average proportions of *S. pombe* reads (untreated = 10.4%, HU-treated = 11.5%) (Fig 2E). Consequently, utilizing the spiked-in reads for read count normalization of wild-type cells had almost no impact on the correlation (r_2_) between the untreated and HU-conditions (S5 Fig).

### HU exposure increased the proportion of downregulated genes in mutants lacking H2A.Z incorporation

**Fig 3:**
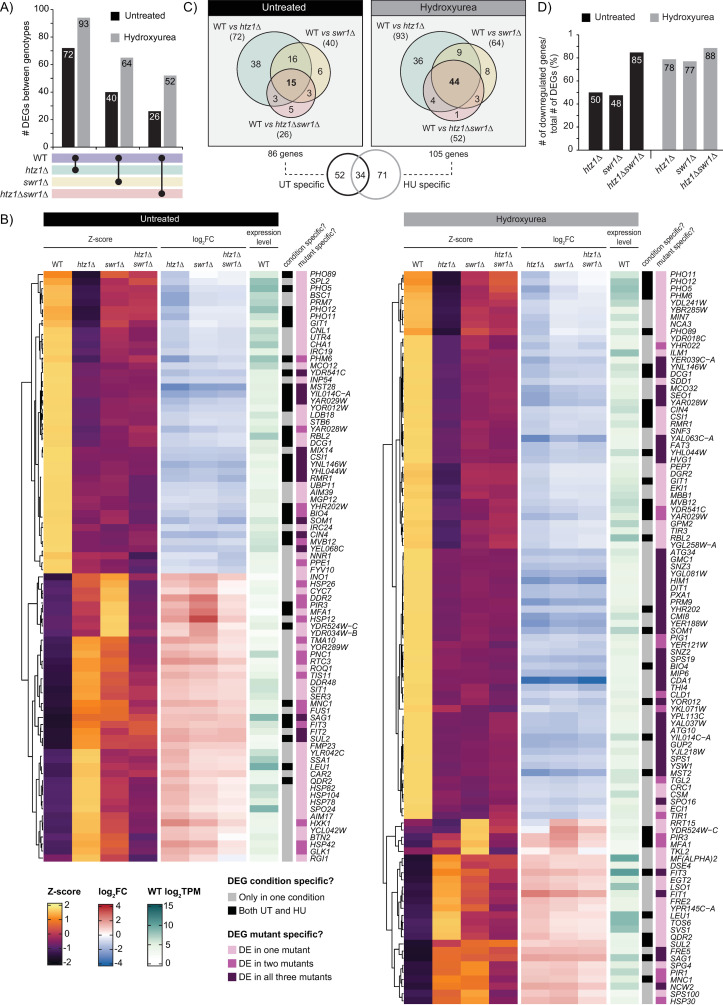
Hydroxyurea exposure increased the proportion of downregulated genes in mutants lacking H2A.Z incorporation. (A) UpSet plot summarizing the number of DEGs identified in each mutant compared to wild-type in the untreated and HU-treated conditions. (B) Heatmaps illustrating the Z-score and log_2_FC of normalized read counts for all the unique DEGs identified between the mutants and wild-type in the untreated condition (86 genes, left) and HU-treated condition (105 genes, right). The average expression level of each gene in wild-type under untreated conditions is presented, along with information on whether the gene was condition specific and the number of mutants in which the gene was identified as differentially expressed. (C) Venn diagram summarizing the number of shared and unique DEGs identified in each mutant compared to wild-type. In the untreated condition, a total of 86 unique genes were found among all three mutants, while 105 unique genes were identified in the HU-treated condition. Among these, 52 were exclusive to the untreated condition, and 71 were specific to the HU-treated condition. (D) Percentage of the DEGs identified for each mutant and condition that were downregulated compared to wild-type.

To determine if H2A.Z incorporation played a role in gene expression regulation in either the untreated or HU-treated conditions, we compared the transcript profiles of the *htz1*Δ, *swr1*Δ, and *htz1*Δ*swr1*Δ mutants to wild-type. Consistent with previous findings [18,22,36–38], relatively few genes were differentially expressed in the absence of H2A.Z incorporation (Fig 3A). In the untreated condition, there were 72 DEGs identified in the *htz1*Δ mutant, 40 in the *swr1*Δ mutant, and 26 in the *htz1*Δ*swr1*Δ mutant. Respectively, these changes amounted to 1.2-0.4% of all genes considered in this study, showing that a remarkably small fraction of genes were significantly perturbed by the absence of H2A.Z incorporation in standard conditions. In agreement with our hypothesis, there was an increase in the number of DEGs identified in the HU-treated condition, with 93 DEGs found in the *htz1*Δ mutant, 64 in the *swr1*Δ mutant, and 52 in the *htz1*Δ*swr1*Δ mutant. Collectively, across all three mutants, we identified 86 genes that were differentially expressed compared to wild-type in the untreated condition and 105 genes in the HU-treated condition (Fig 3B and 3C). For each of these genes, the level of deviation from wild-type varied depending on the genotype. Similar to the PCA results (Fig 1C), the fold change and Z-score for each of the identified DEGs showed that the *htz1*Δ mutant diverged the most from wild-type while the *htz1*Δ*swr1*Δ mutant deviated the least (Fig 3B), which suggested that apo-SWR1-C had an additive detrimental effect on the same genes that were impacted by the loss of H2A.Z incorporation. The patterns of expression for the *swr1*Δ mutant, however, changed depending on if the genes were up or downregulated. While the Z-scores of the *swr1*Δ mutant were indistinguishable from the *htz1*Δ*swr1*Δ mutant for downregulated genes, they were significantly different for upregulated genes (Figs 3B and S6). In this case the *swr1*Δ mutant deviated further from wild-type and even had equivalent Z-scores to the *htz1*Δ mutant for upregulated genes in the HU-treated condition. Therefore, while similar, the *htz1*Δ*, swr1*Δ, and the *htz1*Δ*swr1*Δ mutants did have notable differences in their gene expression profiles.

During our analysis of the DEGs, we found a marked increase in gene overlap between mutants in the HU-treated condition, with 15 genes found in all three mutants in the untreated condition (17% of the DEGs) and 44 genes in the HU-treated condition (42% of DEGs) (Fig 3C). Given that the DEGs in the HU-treated condition could potentially provide insight into the specific role of H2A.Z in the transcriptional response to HU exposure, we looked for shared characteristics between the 44 genes differentially expressed in all three mutants. We found that these genes were not enriched for specific promoter motifs or transcription factors, and their genomic coordinates were evenly distributed across chromosomes and chromosome features (S7A Fig). Furthermore, only two of the genes had a documented sensitivity to HU exposure when deleted (*MST28* and *YGL081W*) (S7B Fig), while Gene Ontology term enrichment identified two terms with weak enrichment: the cell periphery and GMP synthase (S7C Fig). Overall, our analysis found that the DEGs shared by the *htz1*Δ, *swr1*Δ, and *htz1*Δ*swr1*Δ mutants in the HU-treated condition did not represent a coherent functional or regulatory network, which suggested that the loss of H2A.Z incorporation led to broad gene dysregulation that was not specific to a single pathway.

While there were a limited number of genes differentially expressed in the absence of H2A.Z, examining the proportion of DEGs that were downregulated vs upregulated compared to wild-type could provide valuable insight into whether H2A.Z plays a more prominent role in gene activation or repression in *S. cerevisiae*. In the untreated condition both the *htz1*Δ mutant the *swr1*Δ mutant showed an equal number of up and downregulated DEGs (Fig 3D). Conversely, the DEGs identified for the *htz1*Δ*swr1*Δ mutant in the untreated condition were primarily downregulated compared to wild-type (84% of 26 DEGs). In the HU-treated condition, the majority of the DEGs in all three mutants showed lower expression compared to wild-type, with the proportion of downregulated genes in the *htz1*Δ mutant, *swr1*Δ mutant, and *htz1*Δ*swr1*Δ mutant increasing to 78%, 77%, and 88% respectively (Fig 3D). Together, these results suggested that during HU exposure H2A.Z incorporation may play a more prominent role in gene activation than gene repression.

### Apo-SWR1-C contributed to the repression of PHO operon genes

**Fig 4:**
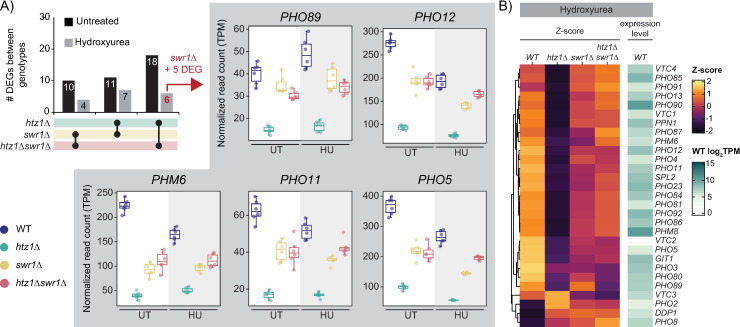
PHO operon genes were repressed in the *htz1*Δ mutant compared to wild-type and the *swr1*Δ and *htz1*Δ*swr1*Δ mutants in both the untreated and HU-treated conditions. (A) Boxplots showing the expression levels of all 5 genes identified as differentially expressed between the *htz1*Δ mutant and *htz1*Δ*swr1*Δ mutant in the HU-treated condition. (B) Heatmaps illustrating the Z-score of normalized read counts for 29 genes in the PHO operon between the mutants and wild-type in the HU-treated condition. The illustrated expression level of each gene is the average in wild-type under HU-treated conditions.

To further characterize the specific effects of apo-SWR1-C on gene expression, we compared the transcript profiles of the *htz1*Δ, *swr1*Δ, and *htz1*Δ*swr1*Δ mutants to one another in both untreated and HU-treated conditions. In general, there were more DEGs identified between each mutant in the untreated condition than the HU-treated condition (Fig 4A). For instance, while 18 genes were differentially expressed between the *htz1*Δ mutant and *htz1*Δ*swr1*Δ mutant in the untreated condition, only six of these genes were identified as differentially expressed in the HU-treated condition, one of which was *SWR1* (Figs 4A and S8). The five other identified genes in the HU-treated condition (*PHO89*, *PHO12*, *PHM6*, *PHO11*, and *PHO5*), were all members of the acid phosphate operon (PHO operon), a set of genes that maintain inorganic phosphate (Pi) homeostasis within the cell during Pi limitation and starvation conditions [55]. In both untreated and HU-treated conditions, these five genes were repressed in the *htz1*Δ mutant compared to wild-type levels. Consistent with the patterns of expression observed in our earlier analysis, the *swr1*Δ and *htz1*Δ*swr1*Δ mutants demonstrated intermediate transcript levels — higher expression than the *htz1*Δ mutant, but lower expression than found in wild-type. This trend was generally conserved across the PHO operon with the *htz1*Δ mutant showing the greatest deviation in expression levels from wild-type in the HU-treated condition for the majority of genes (Fig 4B). Altogether, this suggested that apo-SWR1-C contributed to downregulation of the PHO operon, thus potentially leading to dysregulation of cellular phosphate homeostasis.

### H2A.Z incorporation regulated the expression level of several of the most highly upregulated genes during HU exposure

**Fig 5:**
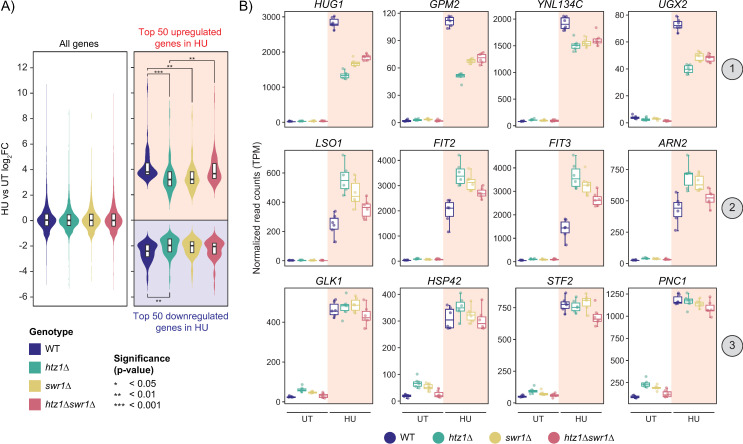
Compared to wild-type, several of the most highly upregulated genes during HU-exposure had smaller fold changes in expression between the untreated and HU-treated conditions in the *htz1*Δ, *swr1*Δ, and *htz1*Δ*swr1*Δ mutants. (A) Violin plots of the log_2_FC between the untreated and HU-treated conditions for all four genotypes. All genes are reported on the left, while the top 50 upregulated genes (red) and top 50 downregulated genes (blue) are visualized on the right. Top genes were determined by their degree of log_2_FC between untreated and HU-treated conditions in wild-type. A one-way ANOVA (α < 0.05) followed by Tukey Kramer post-hoc analysis was used to determine statistically significant comparisons. (B) In the top 50 most upregulated genes in HU, the mutants deviated from wild-type expression in 3 different patterns: 1) similar expression in the untreated condition, but lower expression than wild-type in the HU-treated condition, 2) similar expression in the untreated condition, but higher expression than wild-type in HU-treated condition and 3) higher expression than wild-type in the untreated condition, but similar expression in the HU-treated condition.

Given that *htz1*Δ mutants have defective activation of several inducible genes such as *GAL1*, *PHO5*, and *IMD2* [40–42], we next examined if genes regulated by HU exposure were dependent on H2A.Z for proper activation or repression. To achieve this we compared the expression level fold changes of all four genotypes for the differentially expressed genes that were the most highly upregulated or downregulated during HU exposure in wild-type cells. For the top 50 genes upregulated in HU, wild-type and the *htz1*Δ*swr1*Δ mutant generally had comparable fold changes between the untreated and HU-treated condition, while the *htz1*Δ mutant and the *swr1*Δ mutant had significantly smaller fold changes than wild-type (Fig 5A). Conversely, only the *htz1*Δ mutant showed significantly smaller fold changes for the top 50 genes downregulated in HU. Given that our earlier analysis of DEGs between wild-type and the mutants suggested that H2A.Z played a more prominent role in gene activation, we individually examined the transcript levels of each of the top 50 genes upregulated in HU for all strains in both the untreated and HU-treated condition. Overall, we found that each of the upregulated genes followed one of three patterns of expression: 1) the mutants either exhibited lower expression or 2) higher expression than wild-type in the HU-treated condition, or 3) the mutants displayed higher expression than wild-type in the untreated condition but had comparable levels after HU exposure (Fig 5B). RT-qPCR analysis of representative genes from each of these three categories showed that the trends in expression between each genotype in the HU-treated condition persisted even after the removal of HU, with mRNA levels of each induced gene returning to pre-HU exposure at relatively the same rate in wild-type cells and the *htz1*Δ, *swr1*Δ, and *htz1*Δ*swr1*Δ mutants (S9 Fig). This suggested that H2A.Z incorporation was not required for the reduction of bulk mRNA levels following gene induction. Of all the genes that showed smaller fold changes in the absence of H2A.Z, *HUG1* was particularly noteworthy as, just like HU, its protein product directly binds and inhibits Rnr2, a ribonucleotide reductase that significantly increases in expression during DNA replication stress [56,57] (S10A Fig). Interestingly, many of the genes that showed increased fold changes in the absence of H2A.Z incorporation were involved in the iron regulon (i.e., *FIT2*, *FIT3*, *ARN2*), a pathway that was upregulated in the HU-treated condition [53] (Figs 5B, 2D and S10B). Therefore, while H2A.Z was not required to initiate gene activation in response to HU exposure, its incorporation was necessary for proper wild-type expression of several HU-induced genes.

### Loss of H2A.Z from transcription start sites correlated with, but was not required for, gene induction in response to HU exposure

**Fig 6:**
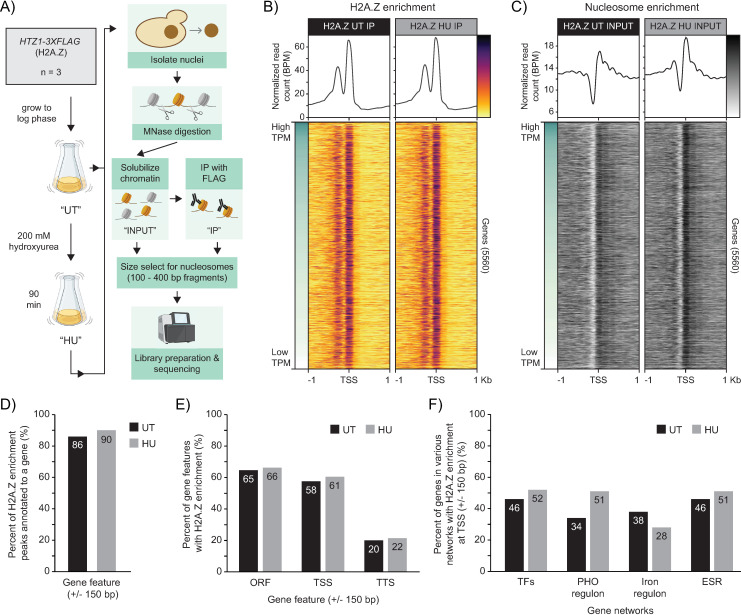
Global H2A.Z enrichment patterns were similar between untreated and HU-treated conditions. (A) MNase-NChIP-seq experimental setup. Graphic created with BioRender.com. (B) Enrichment profiles of H2A.Z and (C) nucleosomes at the TSS of all 5560 genes in untreated and HU-treated cells. Genes in heatmap are sorted by wild-type gene expression level in the untreated condition. (D) Percent of called H2A.Z enrichment peaks that overlapped within 150 bps of a gene feature. A total of 3548 peaks were called in the untreated condition, while 3157 were called in the HU-treated condition. (E) Percent of gene features that are enriched for H2A.Z from the total of 5560 genes used in this study. H2A.Z was considered enriched if a peak called by MACS2 overlapped with the feature coordinates or was within 150 bp of the feature. (F) Percentage of transcription factor (TF) genes and genes part of the iron regulon [53], PHO operon [80], and ESR [52] that were enriched for H2A.Z at their TSS.

Given that the function of H2A.Z in transcription regulation has been tightly linked to its enrichment at TSSs, we next determined if HU exposure affected H2A.Z occupancy or positioning genome-wide. To map H2A.Z incorporation at a single-nucleosome resolution before and after HU exposure, we performed a MNase Native Chromatin Immunoprecipitation of FLAG tagged H2A.Z followed by high-throughput sequencing (MNase-NChIP-seq) of both the solubilized chromatin (INPUT) and H2A.Z enriched (IP) fractions (Figs 6A and S11). When examining the global patterns of H2A.Z enrichment at the TSS of all 5560 genes considered in this study, we found there were minimal differences between the untreated and HU-treated conditions (Fig 6B). Consistent with the previous reports, H2A.Z was highly enriched at the +1 nucleosome in the untreated condition, and there appeared to be no correlation between gene expression level and H2A.Z occupancy [20,22,23]. The HU-treated condition displayed a comparable pattern of H2A.Z enrichment, but with a slightly higher occupancy level at the +1 nucleosome in comparison to the untreated condition. However, this increase was not unique to H2A.Z, as an examination of nucleosome profiles generated from the INPUT fractions revealed an overall rise in nucleosome occupancy at the TSS after HU exposure (Fig 6C). After normalizing H2A.Z enrichment peaks to their respective background nucleosome levels in the INPUT samples, we found H2A.Z was similarly enriched at gene features and pathways in the untreated and HU-treated conditions. The majority of H2A.Z peaks were located within 150 bp of a gene feature (TSS or open reading frames) (86% in untreated, 90% in HU-treated) (Fig 6D), and of the 5560 genes considered in this study, 58% of them had H2A.Z incorporated at their TSS in the untreated condition (61% in the HU-treated condition) (Fig 6E). Furthermore, H2A.Z peaks overlapped with the TSS of numerous genes involved in pathways identified in our mRNA-sequencing analysis, including the ESR, iron regulon and PHO operon (Fig 6F).

**Fig 7:**
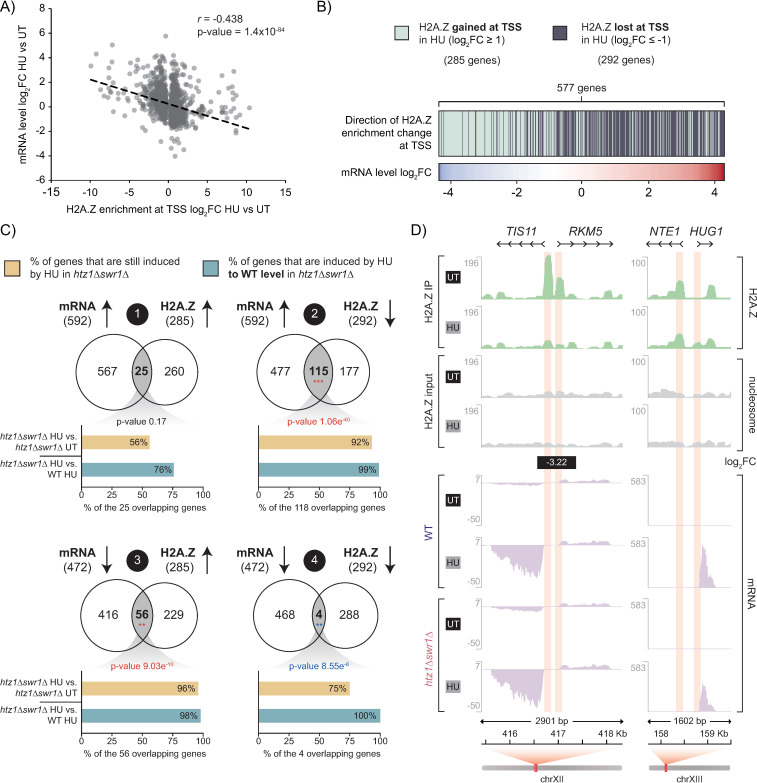
Loss of H2A.Z from transcription start sites was correlated with, but not required for, HU-induced gene activation. (A) Spearman’s correlation coefficient (*r*) between log_2_FC in H2A.Z enrichment at TSS’s (BH-FDR adjusted p-values < 0.05) and log_2_FC of mRNA expression levels of the associated gene. The slope of the correlation is indicated by a dashed black line. (B) 577 genes had changes in H2A.Z enrichment at their TSS in wild-type cells after HU treatment with log_2_FC either ≥ 1 or ≤ −1 (BH-FDR adjusted p-value < 0.05). Genes are ordered by their mRNA level log_2_FC between the untreated and HU-treated conditions in wild-type cells. (C) Overlap between the 1064 DEGs and the 577 genes that exhibited differential H2A.Z enrichment at their TSS during HU-exposure in wild-type cells. There was overlap found in all four groups: 1) genes that had increased mRNA expression and H2A.Z enrichment at their TSS in the HU-treated condition, 2) mRNA expression increased, while H2A.Z enrichment decreased, 3) mRNA expression decreased, while H2A.Z enrichment increased, and 4) genes where mRNA expression and H2A.Z enrichment at the TSS decreased after HU exposure. The p-values, generated using hypergeometric probability, indicate if the overlap in each group was significantly greater (red) or less (blue) than excepted by chance (black = non-significant). The bar plots show the percentage of the overlapping genes in each category that were differentially expressed between the HU-treated *htz1*Δ*swr1*Δ mutant and the UT-treated *htz1*Δ*swr1*Δ mutant (gold), and that were not differentially expressed between the HU-treated *htz1*Δ*swr1*Δ mutant and the HU-treated wild-type (teal). (D) H2A.Z and nucleosome enrichment tracks for *TIS11*, *RKM5, NTE1*, and *HUG1* along with their corresponding transcript tracks untreated and HU-treated conditions. Black box represents the coordinates and log_2_FC of an H2A.Z peak identified as differentially enriched by DiffBind. The pink boxes indicate the position of the +1 nucleosome at the TSS of each gene.

It has previously been proposed that H2A.Z occupancy promotes gene activation by facilitating the disassembly of the +1 nucleosome at TSS [35,58]. To assess if the upregulation of genes during HU treatment was dependent on a loss H2A.Z enrichment at their promoter regions, we examined H2A.Z peaks near TSSs that were differentially enriched between the untreated and HU-treated conditions (Benjamini-Hochberg false discovery rate [BH-FDR] adjusted p-value < 0.05) and matched them with the fold change of expression of their associated gene during HU-exposure (Fig 7A). We found that decreasing H2A.Z enrichment within 150 bp of the TSS was significantly correlated with increased mRNA expression following HU exposure. However, further analysis using the mRNA profiles of the *htz1*Δ*swr1*Δ mutant indicated that while the loss of H2A.Z correlated with an increase in gene expression, a decrease in H2A.Z enrichment at the TSS was generally not required for the proper expression of HU-induced genes. In total 577 genes exhibited differences in H2A.Z enrichment at their TSS upon HU exposure (log_2_ Fold Change [log_2_FC] > 1 or <-1, BH-FDR adjusted p-value < 0.05) (Fig 7B). While 115 of those genes showed both a decrease in H2A.Z enrichment and an increase in mRNA levels after HU-exposure, 92% of these genes were still significantly upregulated by HU in the *htz1*Δ*swr1*Δ mutant (*htz1*Δ*swr1*Δ-HU vs. *htz1*Δ*swr1*Δ-UT), and 99% of them had comparable expression levels between the *htz1*Δ*swr1*Δ mutant and wild-type during HU-exposure (*htz1*Δ*swr1*Δ-HU vs. WT-HU) (Fig 7C). For example, *TIS11*, a gene upregulated by HU that plays a central role in the iron regulon [53], showed a significant decrease in H2A.Z enrichment at its +1 nucleosome in the HU-treated condition, but had comparable transcript levels in both wild-type and the *htz1*Δ*swr1*Δ mutant (Fig 7D). In contrast, another gene highly upregulated by HU-exposure, *HUG1*, which was expressed at a lower level in the *htz1*Δ*swr1*Δ mutant in the HU-treated condition, had minimal H2A.Z incorporation at its transcription start site. Therefore, while HU exposure did affect H2A.Z occupancy at hundreds of genes, changes in H2A.Z enrichment at TSSs was generally not required to induce transcriptional reprogramming during HU exposure.

## Discussion

In this study, we generated a robust gene expression dataset to explore the complex relationship between H2A.Z and gene expression during HU exposure in *S. cerevisiae*. Our findings revealed that H2A.Z incorporation was more prominently involved in gene activation than repression, and that while relatively few genes were differentially expressed in the absence of H2A.Z, its incorporation was necessary for proper wild-type expression of several HU-induced genes. Furthermore, we observed that apo-SWR1-C contributed to the gene expression defects present in the *htz1*Δ mutant, particularly for genes involved in the PHO operon. Additionally, our investigation demonstrated that decreases in H2A.Z enrichment at TSSs correlated with, but was not generally required for, increased mRNA expression of HU-activated genes. Collectively, our results underscore the importance of both accounting for the confounding effects of apo-SWR1-C and using a model that induces widespread transcriptional changes to effectively examine the general role of H2A.Z in gene expression regulation.

Our data provide new insights into how differences in gene expression could contribute to the slow growth phenotypes of the *htz1*Δ, *swr1*Δ, and *htz1*Δ*swr1*Δ mutants during HU exposure. In our analysis we identified several genes and pathways associated with dNTP use and production that were dysregulated in the absence of H2A.Z incorporation. For example, *HUG1*, a ribonucleotide reductase (RNR) inhibitor that is highly upregulated upon DNA replication stress [56,57], exhibited lower transcript levels in the HU-treated condition in all three mutants compared to wild-type. Furthermore, the absence of H2A.Z incorporation resulted in the upregulation of the iron regulon and the repression of several PHO operon genes. Given that the iron regulon optimizes RNR protein function [53,59], and downregulation of PHO operon genes results in reduced nucleotide steady-state levels and *de novo* nucleotide synthesis [60], our results suggest that H2A.Z incorporation could be required for cells to generate and maintain adequate dNTP pools. These pathways are particularly noteworthy as an important part of the transcriptional response to HU is centered around replenishing depleted dNTPs pools and optimizing their usage [45,57]. All together, these differences in expression provide a possible explanation for why the *htz1*Δ, *swr1*Δ, and *htz1*Δ*swr1*Δ mutants are sensitive to HU exposure and DNA replication stress in general.

Here we showed that apo-SWR1-C had a detrimental additive effect on the same genes impacted by the loss of H2A.Z incorporation in both standard and HU-treated conditions. Our results revealed that DEGs exhibited the greatest deviation from wild-type in the *htz1*Δ mutant, with more intermediate expression levels observed in the *swr1*Δ and *htz1*Δ*swr1*Δ mutants. This trend was especially evident for PHO operon genes but was also apparent for the majority of genes that showed decreased expression in the *htz1*Δ mutant. Therefore, while several of the identified DEGs were specific to each genotype, which is consistent with previous work [18,37,61], our results suggest that a shared set of genes were dysregulated across each mutant, differing only in the severity of the expression defect. While both the *swr1*Δ mutant and *htz1*Δ*swr1*Δ mutant generally had indistinguishable gene expression from one another, there were subtle differences that may be attributed to nonspecific interactions of unincorporated H2A.Z with nucleosomes in the *swr1*Δ mutant [62]. However, the expression differences between the *htz1*Δ mutant and the *swr1*Δ and *htz1*Δ*swr1*Δ mutants likely result from deleterious activity of the SWR1-C lacking its H2A.Z substrate. Given that SWR1-C is not dependent on H2A.Z to bind TSSs [63], but H2A.Z is required for its efficient release from chromatin [64], it is likely that the deleterious effects of apo-SWR1-C resulted from a prolonged interaction with chromatin. Coinciding with this idea, *in vitro* experiments have demonstrated that SWR1-C binding is sufficient to initiate, but not complete, DNA unwrapping from nucleosomes [65], which may have transcriptional consequences *in vivo*. Therefore, to effectively examine the effects of H2A.Z incorporation on gene expression in *S. cerevisiae*, it is imperative to consider and address the additive effects of apo-SWR1-C on transcript profiles.

While it is clear that H2A.Z has a diverse role in transcription regulation, and does not adhere to a universal paradigm [3], our results suggest that H2A.Z played a larger role in gene activation than repression in *S. cerevisiae*, particularly during HU exposure. Here we showed that controlling for the additive effects of apo-SWR1-C, or examining gene expression during HU exposure, revealed that the majority of DEGs caused by the absence of H2A.Z incorporation were downregulated compared to wild-type. Furthermore, by comparing gene expression between untreated and HU-treated conditions we showed that several genes highly upregulated during HU-exposure had smaller fold changes in the absence of H2A.Z incorporation. Given that many of the genes induced by HU exposure are also part of the ESR, a general stress response pathway in yeast [52], it is plausible that H2A.Z function is not specific to gene activation during HU exposure, but is instead a broad regulator of gene induction during an array of cellular stress conditions. Our findings are also consistent with previous work in budding yeast showing that many H2A.Z-dependent genes are only dysregulated in *HTZ1* mutants when they are induced or derepressed [40–43,66,67]. Therefore it is likely that results from previous microarray analysis, which found an equal number of upregulated and downregulated genes in the *htz1*Δ mutant, are largely the consequence of using steady-state experimental conditions [18,22,36–38].

The prevailing model for the role of H2A.Z in gene expression regulation is that H2A.Z occupancy promotes gene activation by facilitating the disassembly of the +1 nucleosome, thereby promoting RNAPII initiation and elongation [35,58,68]. However, our results indicate that even when normalized to nucleosome background levels, H2A.Z occupancy at transcription start sites had minimal impact on gene expression in either untreated or HU-treated conditions. H2A.Z-dependent genes appeared to be largely independent of the presence or absence of H2A.Z from their +1 nucleosome. For example, one of the most highly upregulated genes during HU-exposure, *HUG1*, which showed lower expression in the *htz1*Δ*swr1*Δ mutant, had minimal H2A.Z incorporation at its transcription start site. This observation corroborates our previous findings showing that H2A.Z mutants lacking H2A.Z’s specific localization can still recapitulate H2A.Z-dependent gene expression [43]. One potential explanation for our findings is that H2A.Z may influence gene expression indirectly during DNA replication stress through mechanisms completely independent from its localization at the +1 nucleosome. For instance, in addition to depleting dNTP pools, HU exposure increases the production of reactive oxidative species, stalls replication forks, and increases the number of transcription-replication conflicts [44–46], events that all have the potential to disrupt proper chromatin assembly and therefore increase widespread promiscuous transcriptional activity across the genome [69]. In our study this could have led to the identification of DEGs during HU exposure that were unrelated to H2A.Z enrichment at their TSS. However, another possibility is that our experimental conditions were unable to capture the more dynamic aspects of H2A.Z function at the +1 nucleosome in transcription regulation. For instance, H2A.Z may be involved in the rapid transcriptional reprogramming necessary for cells to adapt to the introduction of cellular stress [70], which would not be immediately evident when evaluating bulk mRNA levels 90 minutes after a gene is induced. To address this, future studies could analyze nascent transcripts during genotoxic stress before and after conditional depletion of either H2A.Z or Swr1. This approach could offer deeper insights into the dynamic effects of H2A.Z and SWR1-C on gene expression regulation, independent from the existing pool of mRNA. Nevertheless, our results suggest that H2A.Z was involved in the regulation of the transcriptional response to HU, and its function in gene expression was not entirely dependent on its specific incorporation at transcription start sites.

## Materials and methods

### Yeast strains

Yeast strains used in this study, described in Table 1, were constructed in the *S. cerevisiae* W303 background using standard genetic techniques. Gene deletions were accomplished using the one-step gene replacement method to integrate PCR-amplified segments [71]. Double mutant strains were generated by standard genetic manipulation via mating and tetrad dissection.

**Table 1:** Yeast strains used in this study.

Strain	Genotype	Source
MKY5	*W303*: *MAT*α *leu2-3,112 trp1-1 can1-100 ura3-1 ade2-1 his3-11,15 [phi*+*]*	[67]
MKY1144	MKY5, *htz1*Δ*::HYGMX*	[67]
MKY2101	MKY5, *swr1*Δ*::HIS*	This study
MKY2102	MKY5, *htz1*Δ*::HYGMX, swr1*Δ*::HIS*	This study
MKY353	MKY5, *HTZ1-3×FLAG::KANMX*	This study
JKpX2-8D	*Schizosaccharomyces pombe, leu1-32 his5-303 h*+	[72]

### RNA extraction and sequencing

Saturated overnight cultures were initially diluted to 0.2 OD_600_ in YPD (2% glucose). When the cells reached an OD_600_ 0.5, 7.5 OD units of culture were collected (untreated condition). HU was added to the remaining cells to a final concentration of 200 mM and incubated for 90 min, after which 7.5 OD units of culture were collected (HU-treated condition). To collect samples for both conditions, cells were mixed with methanol (40% final concentration), washed with water, flash frozen in a dry ice bath, and then stored at −80˚C. A total of six biological replicates were independently collected. *Schizosaccharomyces pombe* cells were spiked-in to achieve a 1:10 ratio with the *S. cerevisiae* cells. Total RNA was then extracted and purified using the RNeasy minikit (Qiagen) following the manufacturer’s instructions. The RNA samples’ quality was assessed using a Bioanalyzer (Agilent), which indicated all 48 samples had a RIN value ≥ 8. Libraries were generated using the Illumina TruSeq Stranded mRNA kit and then sequenced with the Illumina NextSeq 500 Mid Output kit (150 cycles).

### RNA-sequencing quality control, alignment, and normalization

The pipeline used for quality control, read alignment, and normalization is summarized in S1A Fig. Read quality was assessed using FastQC (0.11.9). Reads were aligned with the *S. cerevisiae* reference genome (SaCcer3 R64-3-1; www.yeastgenome.org/) using STAR (Version 2.7.7a) [73]. Only uniquely mapping and concordantly aligned reads were retained (MAPQ = 255), leaving 14.1–17.6 million paired-end sequencing reads per sample which gave a minimum genome coverage of 85X. multiBamSummary (deepTools 3.5.4) [74] (Bin size = 100) followed by both principal component analysis (PCA) and Spearman’s correlation coefficients were used to assess the similarity between the expression profiles of each sample. Raw read counts for all annotated genes in the yeast genome (www.yeastgenome.org) were quantified using SeqMonk (Version 1.48.1). The gene list was filtered to remove blacklisted genes which included dubious open reading frames (ORFs), ORFs that were not part of the SacCer3 (R64-3-1) reference genome annotation (R64-1-1.110; www.ensembl.org), and any genes that had average raw read counts less than 10 for all samples (S1A Fig). The raw read counts for the remaining 5560 genes were normalized as Transcripts per Million (TPM).

To examine if total RNA levels varied across the untreated and HU-treated condition, raw read counts in wild-type cells were normalized to the proportion of *Schizosaccharomyces pombe* raw reads present in the library. The normalization factor for each sample was determined using the average library sizes of the cleaned raw read counts for both *S. cerevisiae* and *S. pombe* in the untreated condition:


$$Normalization\;Factor=\frac{S.\;cerevisiae\left(individual\;sample\right)/S.pombe\left(individual\;sample\right)}{S.\;cerevisiae\left(WT\;average\right)/S.pombe\left(WT\;average\right)}$$

Variance (σ^2^) between biological replicates for each strain in each condition was visualized by grouping genes based on wild-type expression levels (TPM) in either the untreated or HU-treated condition. To determine which comparisons were statistically significant in each category we used a one-way ANOVA (α < 0.05) followed by Tukey Kramer post-hoc analysis.

### Differential gene expression analysis and visualization

Differential gene expression analysis was performed using DESeq2 (1.38.3) [51]. Non-normalized raw read counts were used for input as per DESeq2 recommendations. Genes were considered differentially expressed between groups if they met the following criteria: 1) had a Benjamini-Hochberg false discovery rate (BH-FDR) p-value < 0.05, 2) either a log_2_FC ≥ 1 or ≤ −1, and 3) if the gene had a log_2_TPM value ≥ 3.5 in at least one of the two compared samples. The ComplexHeatmap (2.14.0) R-package was used to create heatmaps visualizing log_2_FC [75]. Functional enrichment analysis of differentially expressed genes was performed using g:Profiler (Version e110_eg57_p18_4b54a898) and the platform's recommended g:SCS multiple testing correction method [76] with a significance threshold of 0.05. Chromosome locations of genes were visualized using RIdeogram (0.2.2) [77].

### Growth assays

Overnight cultures grown in YPD (2% glucose) were diluted to 0.5 OD_600_. Cells were 10-fold serially diluted and spotted onto YPD plates with or without a final concentration of 125 mM HU, 0.015% methyl methanesulfonate (MMS), and 15 μg/mL benomyl. Plates were incubated at 30˚C for 3 days. The assay was performed for three biological replicates.

### Analysis of cell cycle by flow cytometry

Cell cycle profiles for three biological replicates were obtained by measuring cellular DNA content using flow cytometry [78]. In brief, 1 OD unit of untreated and HU-treated cell cultures were fixed with 95% ethanol and stored at −20˚C overnight. Ethanol was removed and cells resuspended in 50 mM sodium citrate buffer containing 20 μg/mL RNase A and 2.5 μM Sytox Green (Molecular Probes S7020). Samples were then placed at 37˚C for 1 hour, followed by a proteinase K digestion for 1 hour at 55˚C. Samples were incubated overnight at 4˚C. Cell suspensions were sonicated for 30 seconds immediately prior to flow cytometry. Sytox green fluorescence was obtained for 50, 000 cells on a LSRFortessa X-20 flow cytometer using Diva 8 acquisition software. Cells were gated using FSC area and SSC area to distinguish cells from debris, and Syotx green width by Sytox green area to distinguish single cells from doublets. Automated quantification of the DNA content histograms was performed using FlowJo 10.10.

### RT-qPCR

Overnight yeast cultures were diluted to 0.2 OD600 in YPD. When the cells reached an OD_600_ 0.5, 7.5 OD units of culture were collected (untreated condition). HU was added to the remaining cells to a final concentration of 200 mM and incubated for 90 min, after which 7.5 OD units of culture were collected (HU-treated condition). Cells were washed and then resuspended in fresh YPD. Samples were collected 30 minutes, 60 minutes, and 90 minutes after being placed in fresh media. RNA was extracted using the RNeasy Mini Kit (Qiagen) and converted to cDNA using the QuantiTect Reverse Transcription Kit (Qiagen). cDNA was analyzed using a Rotor-Gene 6000 (Corbett Research) and PerfeCTa SYBR green FastMix (Quanta Biosciences). Samples were analyzed from three independent biological replicates and normalized to both *ALG9* mRNA levels and to the median wild-type mRNA levels in the untreated condition.

### MNase-NChIP-seq library generation and sequencing

The MNase Native Chromatin Immunoprecipitation (MNase-NChIP) protocol (summarized in Fig 6A), was performed as previously described [79] for three biological replicates. In brief, saturated overnight yeast cultures were back diluted to 0.2 OD_600_ and then grown to 0.6 OD_600_ in 500 mL of YPD (2% glucose). 250 mL of culture was collected before (untreated) and after incubation with 200 mM HU for 90 minutes (HU-treated). In order to isolate nuclei, cells were treated with 25 ul of 20 mg/mL T100 Zymolyase and incubated for 10 minutes at 37°C. The isolated nuclei were digested with 5 U/ml of MNase for 30 minutes at 37°C and then solubilized by passing through a 20-G and 26-G needle four times each. 100 µL of the solubilized chromatin was set aside for the DNA INPUT control, while the remaining sample was incubated with anti-FLAG antibody (40 µg) (Sigma, F3165) coupled to 100 µL of Protein A Dynabeads (Invitrogen) for 16 hours (the H2A.Z immunoprecipitation [IP] sample). DNA fragments were purified from the IP and INPUT samples and underwent size selection using SPRI beads to obtain fragments between 100–400 bps in size. Libraries were prepared using a NEB DNA Ultra II Prep kit and then sequenced with the Illumina NextSeq 100 cycles P1 kit (100 million PE reads).

### H2A.Z and nucleosome enrichment quality control and visualization

FASTQC was used to confirm the quality of the raw reads (0.12.0). Reads were aligned to the *S. cerevisiae* reference genome (SaCcer3 R64-3-1; www.yeastgenome.org/) using BWA-mem. Only uniquely mapping and concordantly aligned reads were retained (MAPQ = 60). The deepTools suite (3.5.4) was used to assess the enrichment signal of each sample. We used multiBamSummary (Bin size = 100) followed by both plotCorrelation and plotPCA as well as plotFingerprint to assess the similarity between the enrichment profiles of each sample and determine if the H2A.Z signal in the IP samples could be differentiated from background (INPUT samples), respectively. To visualize H2A.Z and nucleosome enrichment across the genome, bamCoverage was used to create bigWigs using the following parameters: normalized as bin per million (BPM), paired-end read-extension, keep duplicates, mapping quality minimum of 60, and fragment lengths between 130–200 bp. The three biological replicates for each sample were merged into one bigWig using the SAMtools (1.17) merge function. Enrichment tracks were visualized using IGV (2.8.12). H2A.Z and nucleosome enrichment patterns at TSSs were plotted with deeptools heatmaps using *S. cerevisiae* gene annotation coordinates (R64-1-1.110; www.ensembl.org).

### Identification of differentially enriched H2A.Z peaks

MACS2 (version 2.2.7.1) was used to determine the location and size of H2A.Z enrichment peaks. Matched inputs were used as controls for each individual replicate and sample type. The effective genome size was set to 10.9 Mb (90% of the total genome) to account for the approximately 10% of the genome that produced either suboptimal read quality or low sequence coverage in the MNase-NChIP sequencing data due to repetitive sequences (rDNA) or tightly packed heterochromatin (telomers, centromeres, and mating loci). To focus the analysis on single nucleosomes, the medium fragment size was set to 150 bp, and only fragments greater than 130 bp were considered. Peak detection was multiple test corrected using a BH-FDR of 0.05. Bedtools (2.31.0) intersect was used to create a consensus file of peak locations for each IP sample, such that only the peak coordinates that overlapped in all three biological replicates were retained. Peaks were annotated to the 5560 genes used in the expression analysis. Peaks were considered at the TSS if their genomic coordinate was within 150 bp of the TSS. DiffBind (3.17) was used to determine if the magnitude of any of the called H2A.Z peaks were significantly different between the untreated and HU-treated conditions. Peaks called from each individual replicate, along with their matched INPUT files, were used in the analysis. Peak detection was multiple test corrected using a BH-FDR of 0.05.

## Supporting information

S1 Fig RNA-sequencing quality control, alignment, and normalization. (A) Overview of the pipeline used to generate the normalized read counts from the mRNA libraries. Blacklisted genes (as described in the blue box) were removed leaving a total of 5560 genes for downstream data analysis. (B) Normalized read counts of *HTZ1* and *SWR1* confirmed that the genes were correctly knocked out in the *htz1*Δ, *swr1*Δ, and *htz1*Δ*swr1*Δ mutants.
(TIF)

S2 Fig The cycle profiles of wild type cells and the *htz1*Δ, *swr1*Δ, and *htz1*Δ*swr1*Δ mutants were similar in both the untreated and HU-treated conditions. (A) Variance (σ^2^) between biological replicates for each strain in each condition was visualized by grouping genes based on the quantile of their expression level (TPM) in wild-type: very high (>90 quantile), high (70-90 quantile), medium (30-70 quantile), low (10-30 quantile), very low (<10 quantile). A one-way ANOVA (α < 0.05) followed by Tukey Kramer post-hoc analysis was used to determine which comparisons were statistically significant in each category. (B) The partial rescue of the *htz1*Δ mutant growth defect by simultaneous deletion of *SWR1* was most evident during HU exposure compared to methyl methanesulfonate (MMS) or benomyl conditions. Cells were 10-fold serially diluted, spotted onto YPD media with the indicated concentrations of HU, MMS, and benomyl and grown for three days. (C) Cell cycle profiles obtained by flow cytometry of the wild-type, *htz1*Δ, *swr1*Δ, and *htz1*Δ*swr1*Δ mutants showed no substantial differences in either the untreated or HU-treated conditions.
(TIF)

S3 Fig Thresholds used for determining differentially expressed genes. (A) Distribution plot showing that the raw reads counts of the mRNA transcripts matched DESeq2’s fitted model. (B) Histogram of the average log_2_TPM of each gene in wild-type cells in the untreated condition. If genes had a log_2_TPM < 3.5 in both the untreated and HU-treated condition, they were not classified as differentially expressed, regardless of if they met all other criteria. (C) To ensure that these thresholds were sufficient to filter out genes with biologically insignificant changes in expression, we examined the transcript levels of four genes previously shown to exhibit minimal gene expression changes in various conditions (*TAF10*, *ALG9*, *TFC1*, and *UBC6*) [81] as negative controls, and four genes known to be highly upregulated after HU exposure (*HUG1*, *AHP1*, *RNR2*, and *DDR48*) [47] as positive controls. As expected, the positive control genes were identified as differentially expressed, whereas the negative control genes were not.
(TIF)

S4 Fig Gene Ontology term enrichment analyses of all upregulated DEGs in HU. BP = Biological Process, MF = Molecular function.
(TIF)

S5 Fig S. pombe spike-in normalization did not affect the correlation between the untreated and HU-treated wild-type groups. TPM correlation scatter plots of wild-type untreated and HU-treated cells with and without *S. pombe* spike-in normalization. Spike-in normalization did not correct the skewed slope between the conditions, which could be primarily attributed to a cluster of highly expressed genes that were repressed in the presence of HU (indicated by the red circle). Notably many of the genes in this circle are related to ribosome production and regulation.
(TIF)

S6 Fig The patterns of expression for the *swr1*Δ mutant changed depending on if the genes were upregulated or downregulated. Violin plots of normalized read count Z-scores for all the unique DEGs identified in Fig 3 (expect for *SWR1* and *HTZ1*) between the mutants and wild-type in the untreated condition (84 genes) and HU-treated condition (103 genes), split into up and downregulated genes. The number of genes in each of the four categories is indicated by “†”. A Compact Letter Display is used to indicate the results of all pairwise comparisons among each genotype within each category. Genotypes that share a letter are not-significantly different as determined by a one-way ANOVA (α < 0.05) followed by Tukey Kramer post-hoc analysis. The *swr1*Δ mutant matched with the *htz1*Δ*swr1*Δ mutant for downregulated genes, was the same as the *htz1*Δ mutant in the upregulated HU-treated category, and was significantly different from all strains in the upregulated untreated category.
(TIF)

S7 Fig The DEGs identified in the HU-treated condition were not functionally related. (A) The 44 DEGs that overlapped between all mutants in the HU-condition were evenly distributed across chromosomes (black circles). Locations of the autonomously replicating sequences (ARS) are indicated by the thick black lines. (B) Of the 44 DEGs that overlapped between all mutants in the HU-condition, only two of them matched with the 1095 genes in yeast known to exhibit sensitivity to HU when mutated (as reported by SGD, www.yeastgenome.org). Additionally, five of the DEGs that were exclusive to the *htz1*Δ mutant overlapped with this list. (C) Gene ontology and transcription factor enrichment analyses of the 44 overlapping DEGs in HU. CC = cellular component, MF = Molecular function.
(TIF)

S8 Fig 18 genes were differentially expressed between the htz1Δ mutant and htz1Δswr1Δ mutant in the untreated condition. The expression levels of the 12 genes that were only differentially expressed in the untreated condition are illustrated on the left, while the 6 genes differentially expressed in both the untreated and hydroxyurea condition are on the right (all but *SWR1* are also shown in Fig 4A).
(TIF)

S9 Fig The mRNA levels of H2A.Z-dependent genes highly induced by HU exposure, decreased after HU removal at the same rate in wild-type, and the *htz1*Δ, *swr1*Δ, and *htz1*Δ*swr1*Δ mutants. RT-qPCR analysis of *HUG1*, *FIT3*, and *PNC1* mRNA levels from three replicates were normalized to both *ALG9* mRNA levels and to the median wild-type untreated mRNA levels. RNA was extracted from untreated cells (UT), HU-treated cells (HU), and from cells 30, 60, and 90 minutes after HU-removal.
(TIF)

S10 Fig Ribonucleotide reductases and iron regulon genes were misregulated in the *htz1*Δ, *swr1*Δ, and *htz1*Δ*swr1*Δ mutants compared to wild-type. (A) *RNR1-4* expression levels in the untreated and HU-treated condition for all four genotypes. (B) Heatmaps illustrate the log_2_FC of gene expression between the mutants and wild-type in the HU-treated condition for 29 genes in the iron regulon. The average expression level of each gene in wild-type under untreated conditions is presented on the right.
(TIF)

S11 Fig Quality control for MNase-NChIP-seq experiment. (A) The addition of a 3X-FLAG tag to H2A.Z did not affect cellular growth in HU. Cells were 10-fold serially diluted, spotted onto YPD media with 125 mM HU. (B) Fingerprint plots showing that H2A.Z signal in the IP samples could be successfully differentiated from background signal (INPUT samples). (B) Biplot of PC1 and PC2 generated from a Principal Component Analysis (PCA) of aligned reads. Each data point represents a single biological replicate (n = 3). (C) Spearman’s correlation coefficient matrix of aligned reads from H2A.Z IP and INPUT samples show that samples clustered by condition.
(TIF)

S1 Table Numerical data and gene lists for all graphs. (XLSX)

S2 Table DESeq output data for all comparisons. (XLSX)
