# Supplementary figures and images for: Characterizing the regulatory effects of H2A.Z and SWR1-C on gene expression during hydroxyurea exposure in Saccharomyces cerevisiae
Source: PLoS Genet. 2025 Jan 21;21(1):e1011566. doi: 10.1371/journal.pgen.1011566 (PMC11761084; doi:10.1371/journal.pgen.1011566)

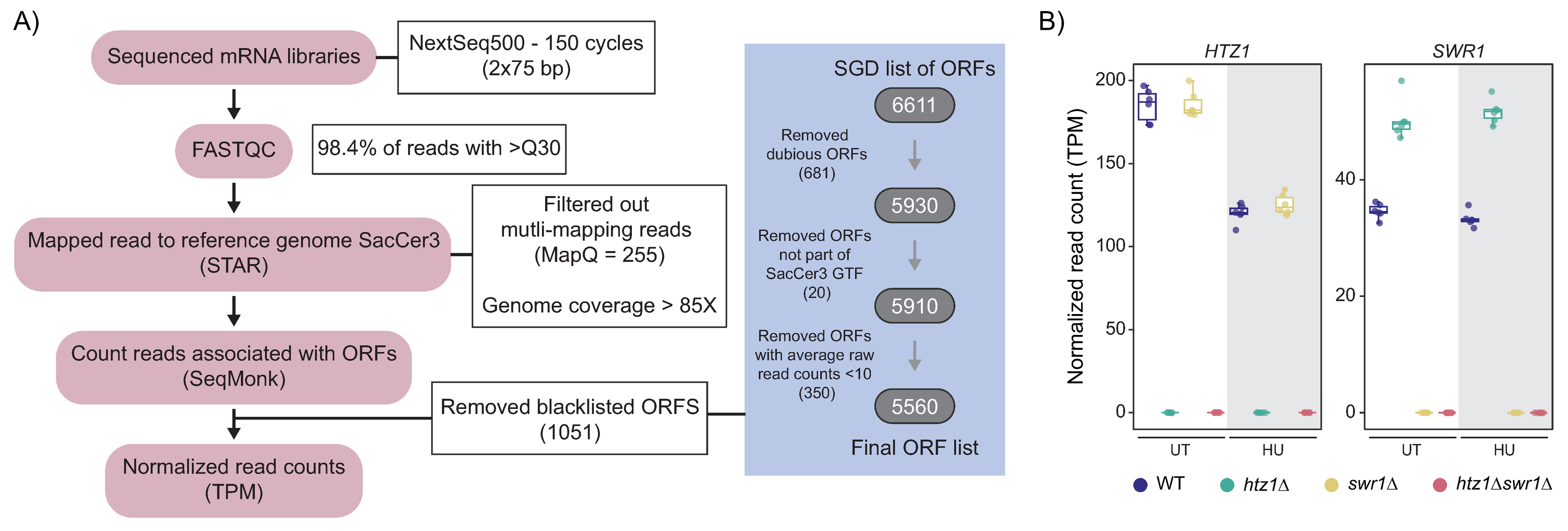

Supplement: S1 Fig — (A) Overview of the pipeline used to generate the normalized read counts from the mRNA libraries. Blacklisted genes (as described in the blue box) were removed leaving a total of 5560 genes for downstream data analysis. (B) Normalized read counts of HTZ1 and SWR1 confirmed that the genes were correctly knocked out in the htz1Δ, swr1Δ, and htz1Δswr1Δ mutants. (TIF) [file pgen.1011566.s001.tif]

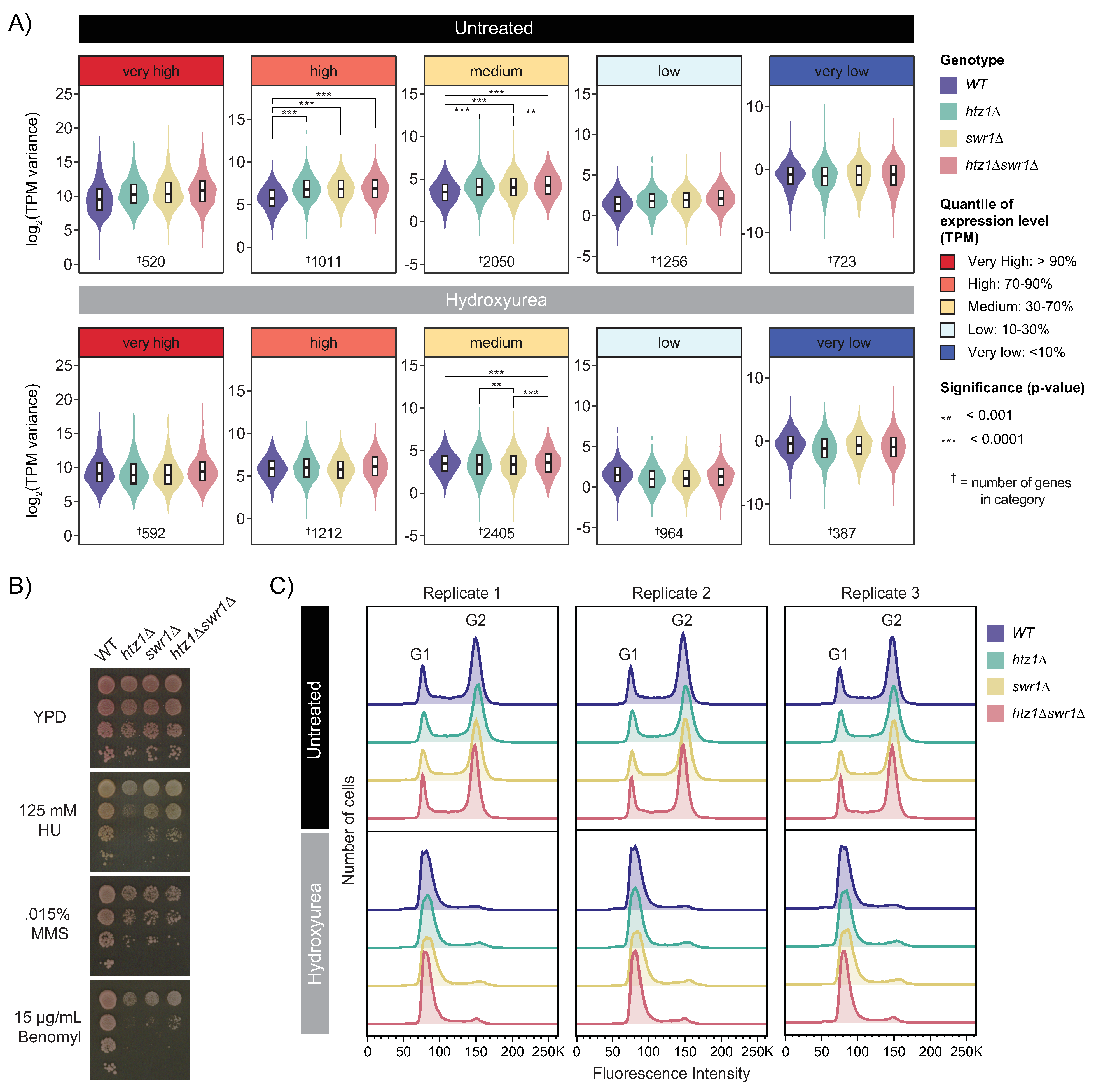

Supplement: S2 Fig — (A) Variance (σ2) between biological replicates for each strain in each condition was visualized by grouping genes based on the quantile of their expression level (TPM) in wild-type: very high (>90 quantile), high (70-90 quantile), medium (30-70 quantile), low (10-30 quantile), very low (<10 quantile). A one-way ANOVA (α < 0.05) followed by Tukey Kramer post-hoc analysis was used to determine which comparisons were statistically significant in each category. (B) The partial rescue of the htz1Δ mutant growth defect by simultaneous deletion of SWR1 was most evident during HU exposure compared to methyl methanesulfonate (MMS) or benomyl conditions. Cells were 10-fold serially diluted, spotted onto YPD media with the indicated concentrations of HU, MMS, and benomyl and grown for three days. (C) Cell cycle profiles obtained by flow cytometry of the wild-type, htz1Δ, swr1Δ, and htz1Δswr1Δ mutants showed no substantial differences in either the untreated or HU-treated conditions. (TIF) [file pgen.1011566.s002.tif]

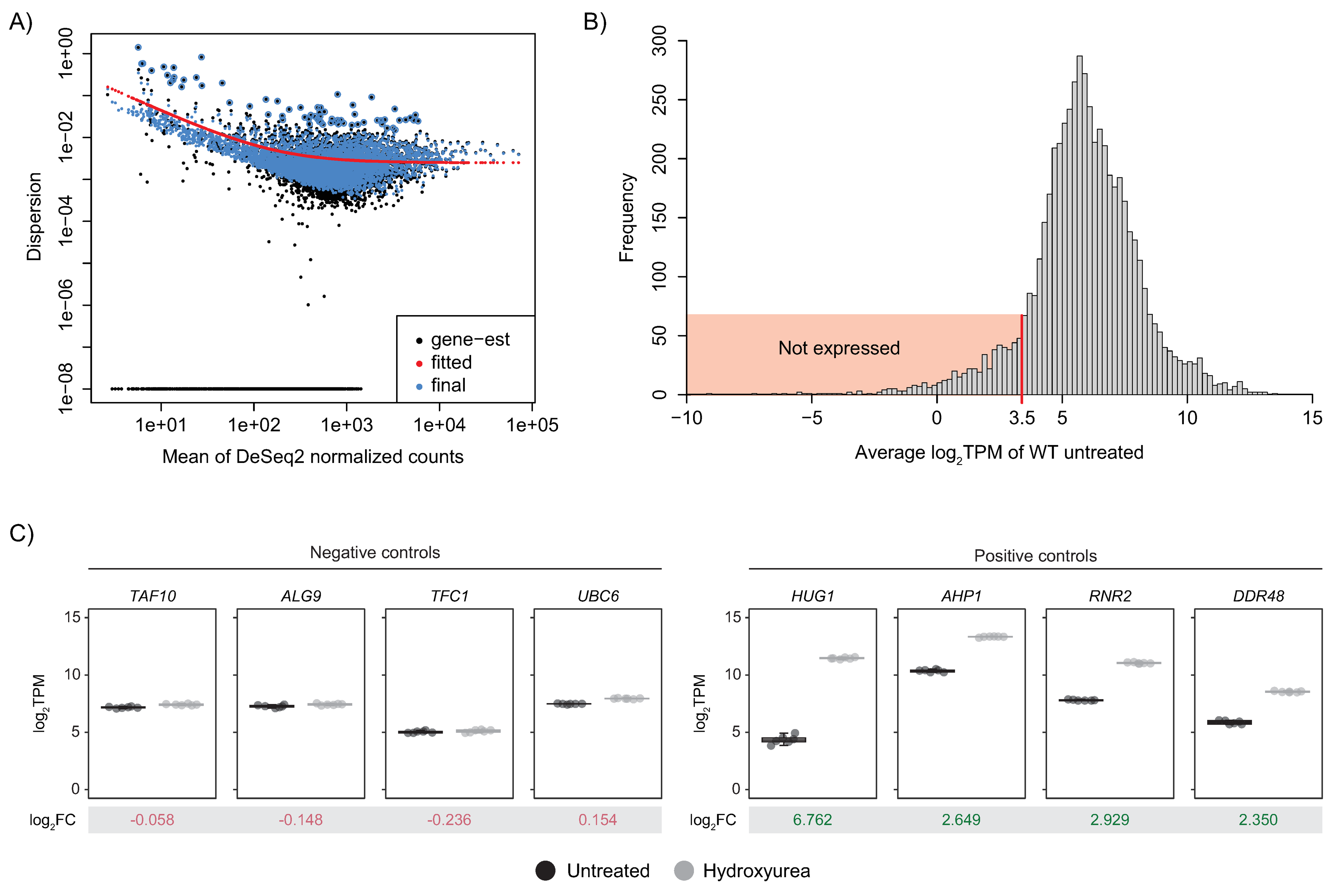

Supplement: S3 Fig — (A) Distribution plot showing that the raw reads counts of the mRNA transcripts matched DESeq2’s fitted model. (B) Histogram of the average log2TPM of each gene in wild-type cells in the untreated condition. If genes had a log2TPM < 3.5 in both the untreated and HU-treated condition, they were not classified as differentially expressed, regardless of if they met all other criteria. (C) To ensure that these thresholds were sufficient to filter out genes with biologically insignificant changes in expression, we examined the transcript levels of four genes previously shown to exhibit minimal gene expression changes in various conditions (TAF10, ALG9, TFC1, and UBC6) [81] as negative controls, and four genes known to be highly upregulated after HU exposure (HUG1, AHP1, RNR2, and DDR48) [47] as positive controls. As expected, the positive control genes were identified as differentially expressed, whereas the negative control genes were not. (TIF) [file pgen.1011566.s003.tif]

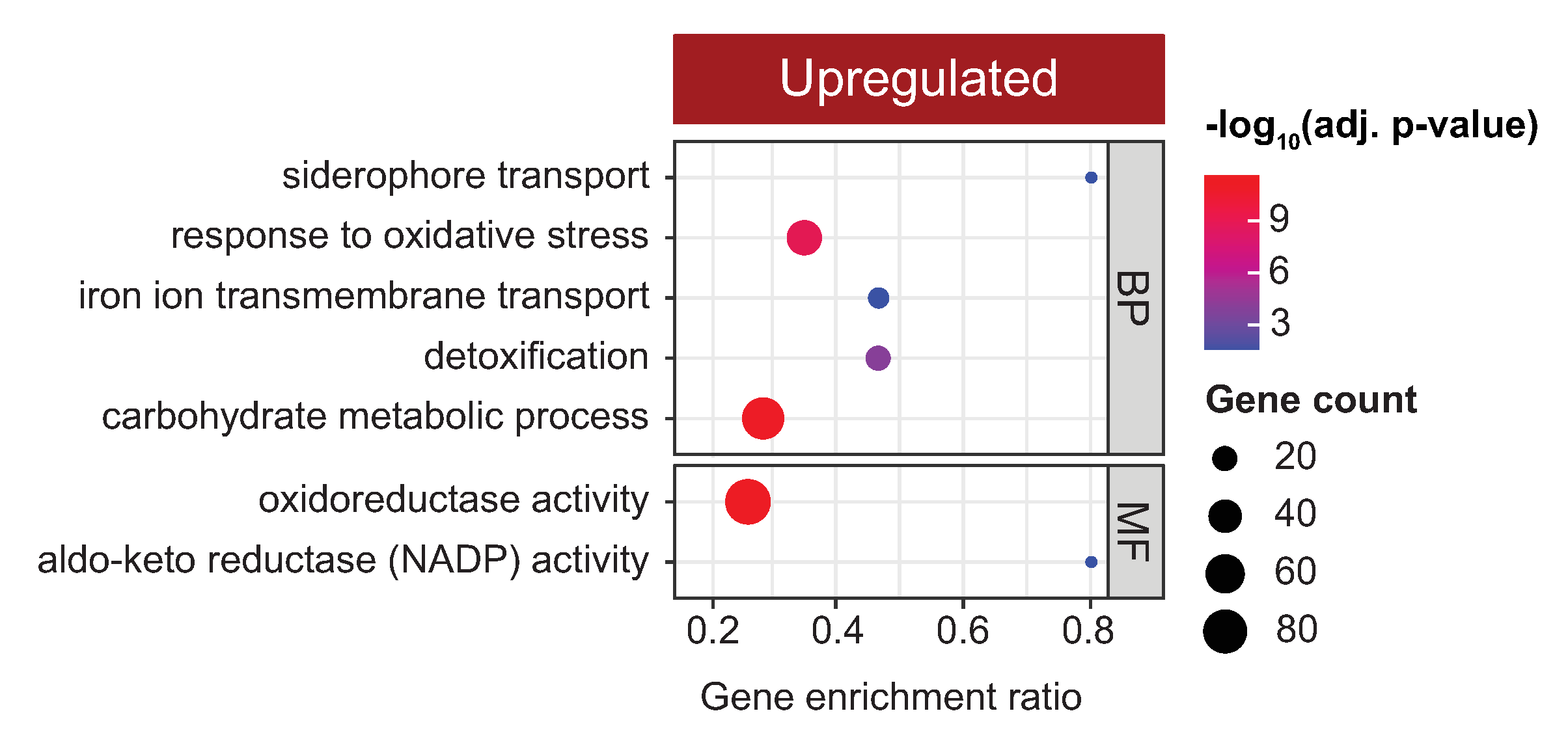

Supplement: S4 Fig — BP = Biological Process, MF = Molecular function. (TIF) [file pgen.1011566.s004.tif]

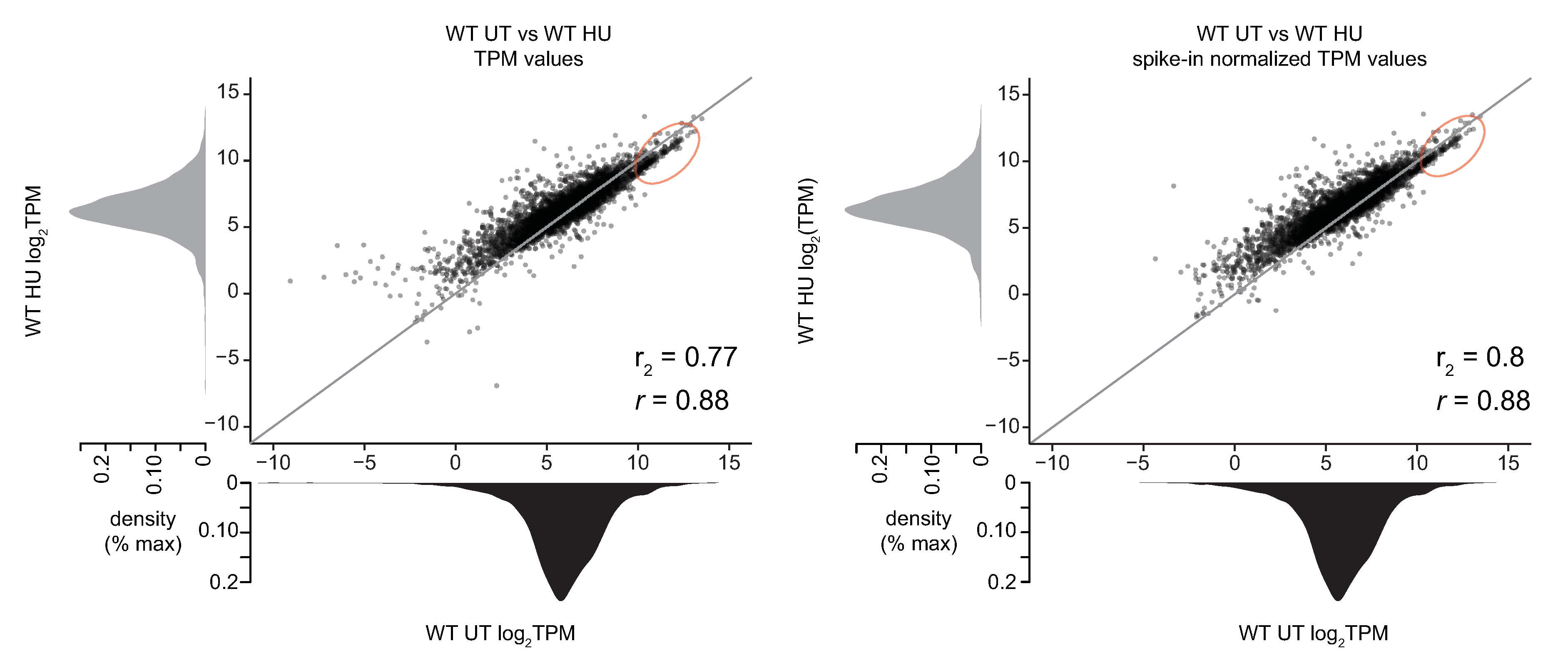

Supplement: S5 Fig — TPM correlation scatter plots of wild-type untreated and HU-treated cells with and without S. pombe spike-in normalization. Spike-in normalization did not correct the skewed slope between the conditions, which could be primarily attributed to a cluster of highly expressed genes that were repressed in the presence of HU (indicated by the red circle). Notably many of the genes in this circle are related to ribosome production and regulation. (TIF) [file pgen.1011566.s005.tif]

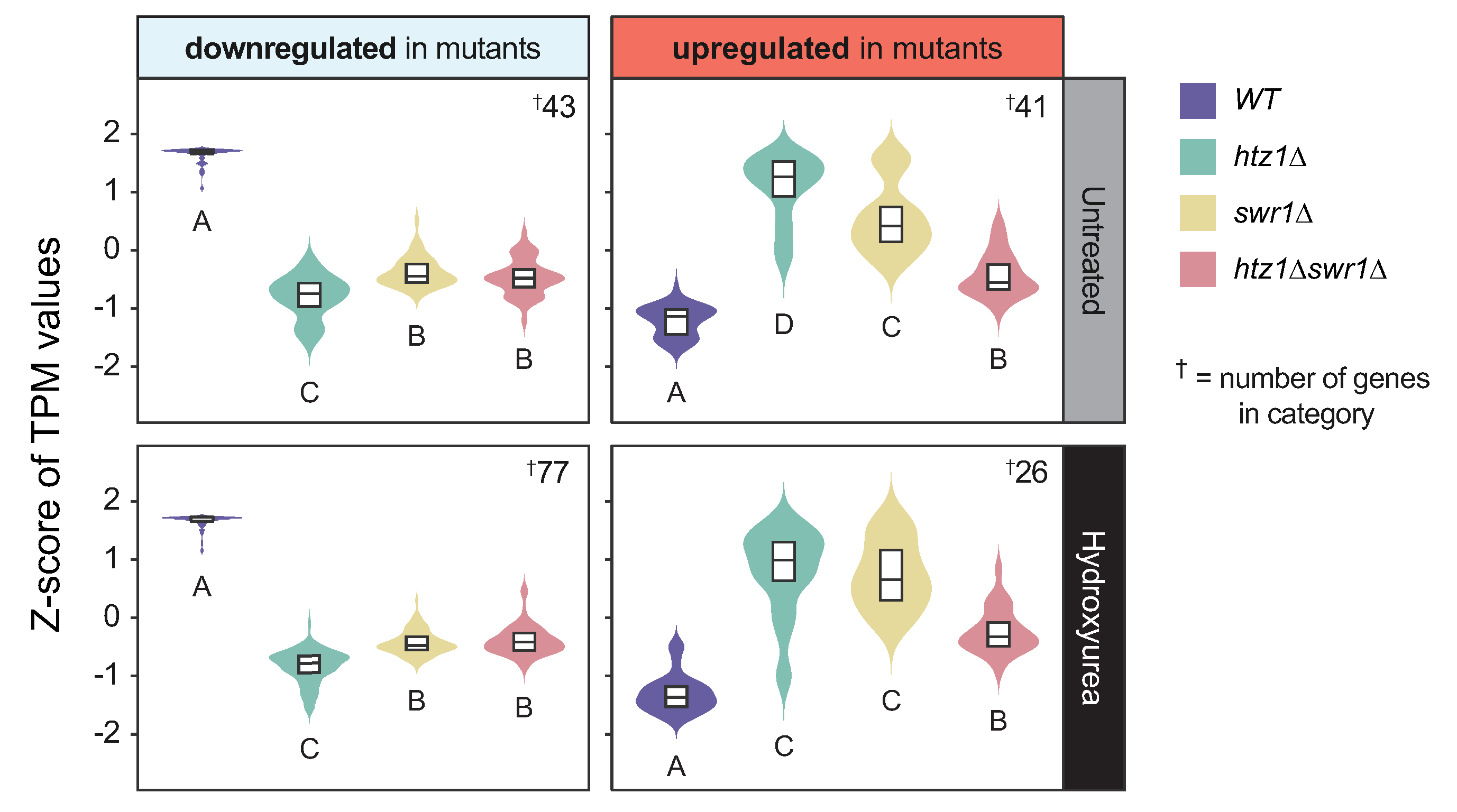

Supplement: S6 Fig — Violin plots of normalized read count Z-scores for all the unique DEGs identified in Fig 3 (expect for SWR1 and HTZ1) between the mutants and wild-type in the untreated condition (84 genes) and HU-treated condition (103 genes), split into up and downregulated genes. The number of genes in each of the four categories is indicated by “†”. A Compact Letter Display is used to indicate the results of all pairwise comparisons among each genotype within each category. Genotypes that share a letter are not-significantly different as determined by a one-way ANOVA (α < 0.05) followed by Tukey Kramer post-hoc analysis. The swr1Δ mutant matched with the htz1Δswr1Δ mutant for downregulated genes, was the same as the htz1Δ mutant in the upregulated HU-treated category, and was significantly different from all strains in the upregulated untreated category. (TIF) [file pgen.1011566.s006.tif]

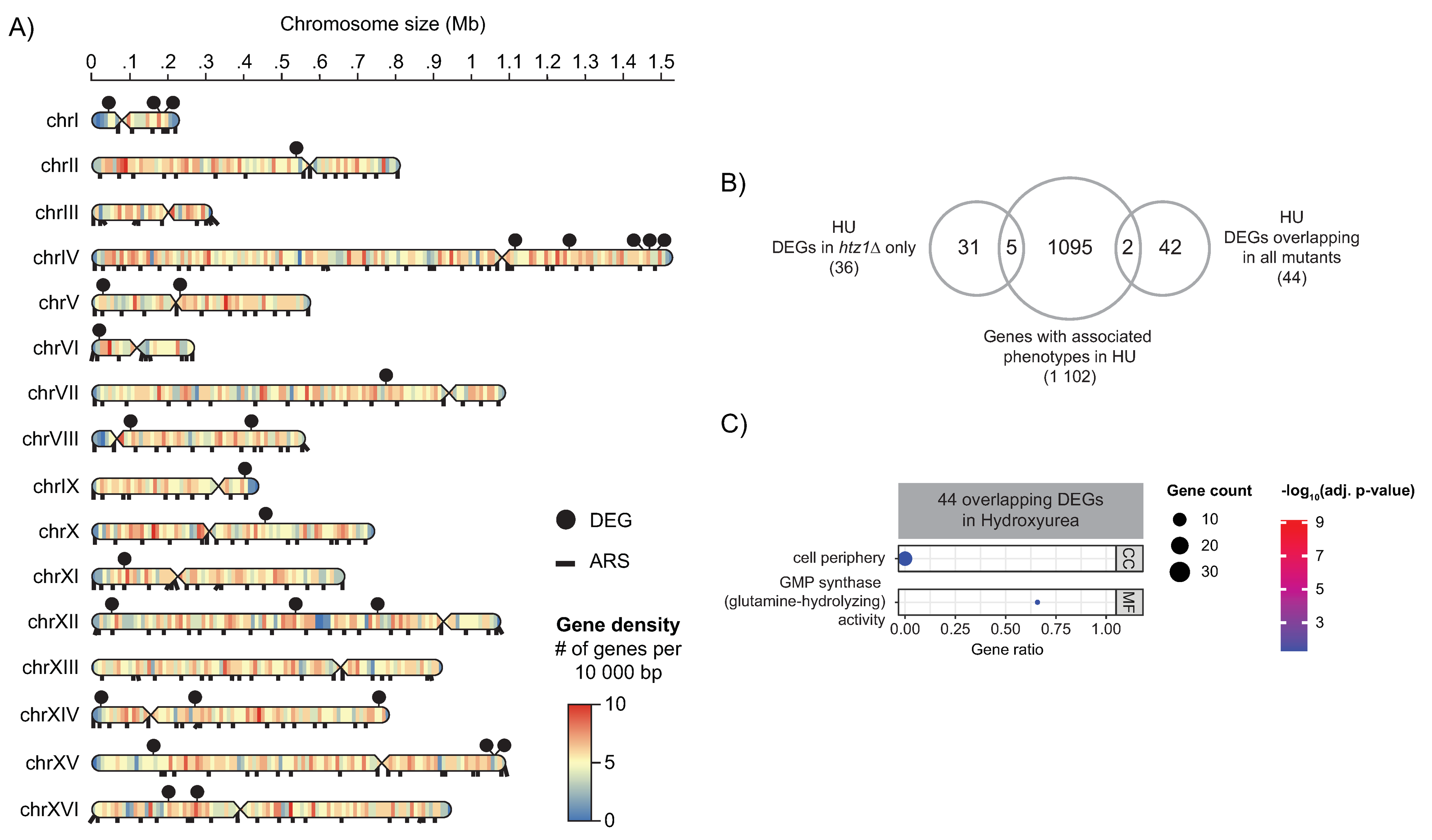

Supplement: S7 Fig — (A) The 44 DEGs that overlapped between all mutants in the HU-condition were evenly distributed across chromosomes (black circles). Locations of the autonomously replicating sequences (ARS) are indicated by the thick black lines. (B) Of the 44 DEGs that overlapped between all mutants in the HU-condition, only two of them matched with the 1095 genes in yeast known to exhibit sensitivity to HU when mutated (as reported by SGD, www.yeastgenome.org). Additionally, five of the DEGs that were exclusive to the htz1Δ mutant overlapped with this list. (C) Gene ontology and transcription factor enrichment analyses of the 44 overlapping DEGs in HU. CC = cellular component, MF = Molecular function. (TIF) [file pgen.1011566.s007.tif]

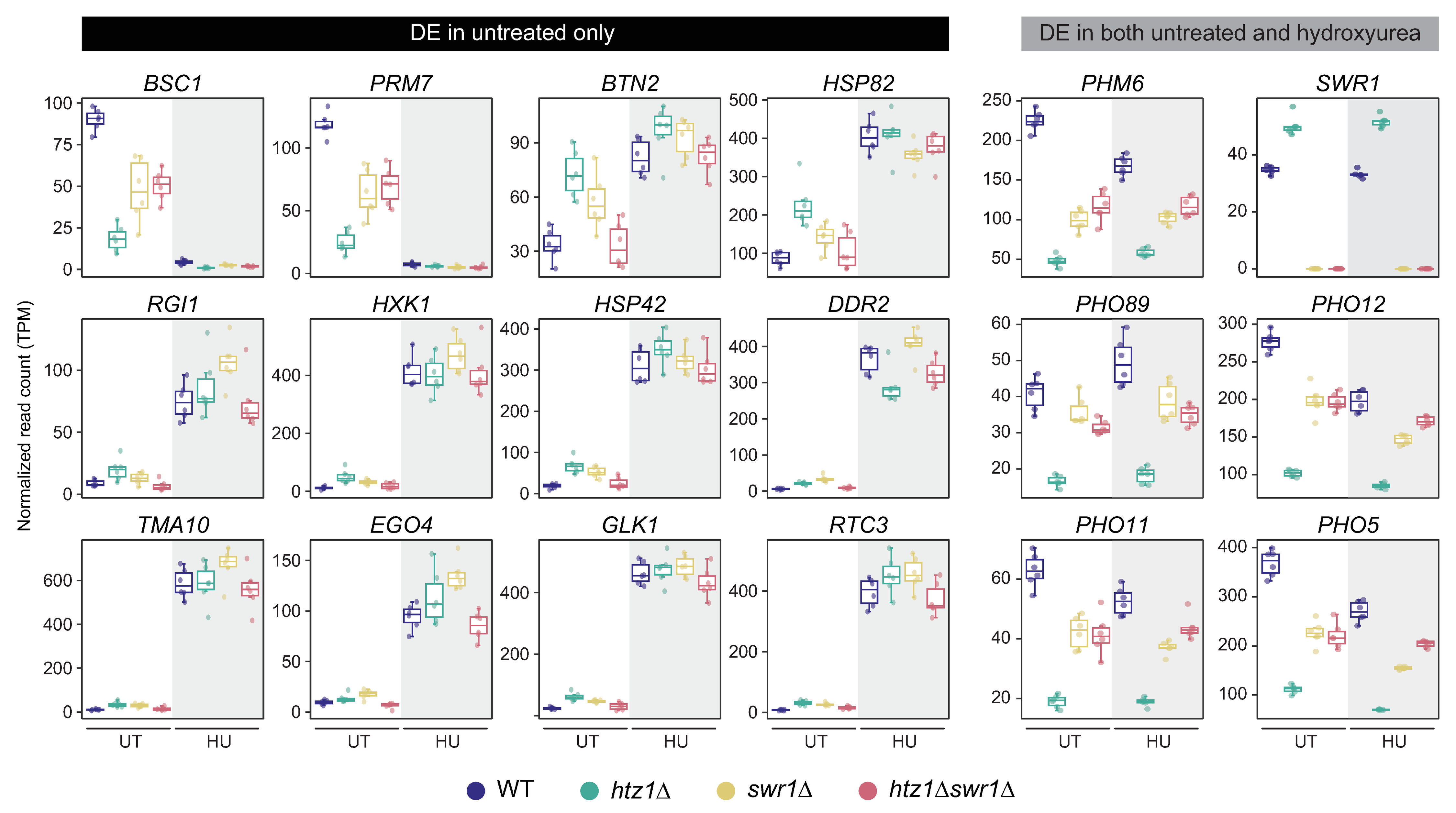

Supplement: S8 Fig — The expression levels of the 12 genes that were only differentially expressed in the untreated condition are illustrated on the left, while the 6 genes differentially expressed in both the untreated and hydroxyurea condition are on the right (all but SWR1 are also shown in Fig 4A). (TIF) [file pgen.1011566.s008.tif]

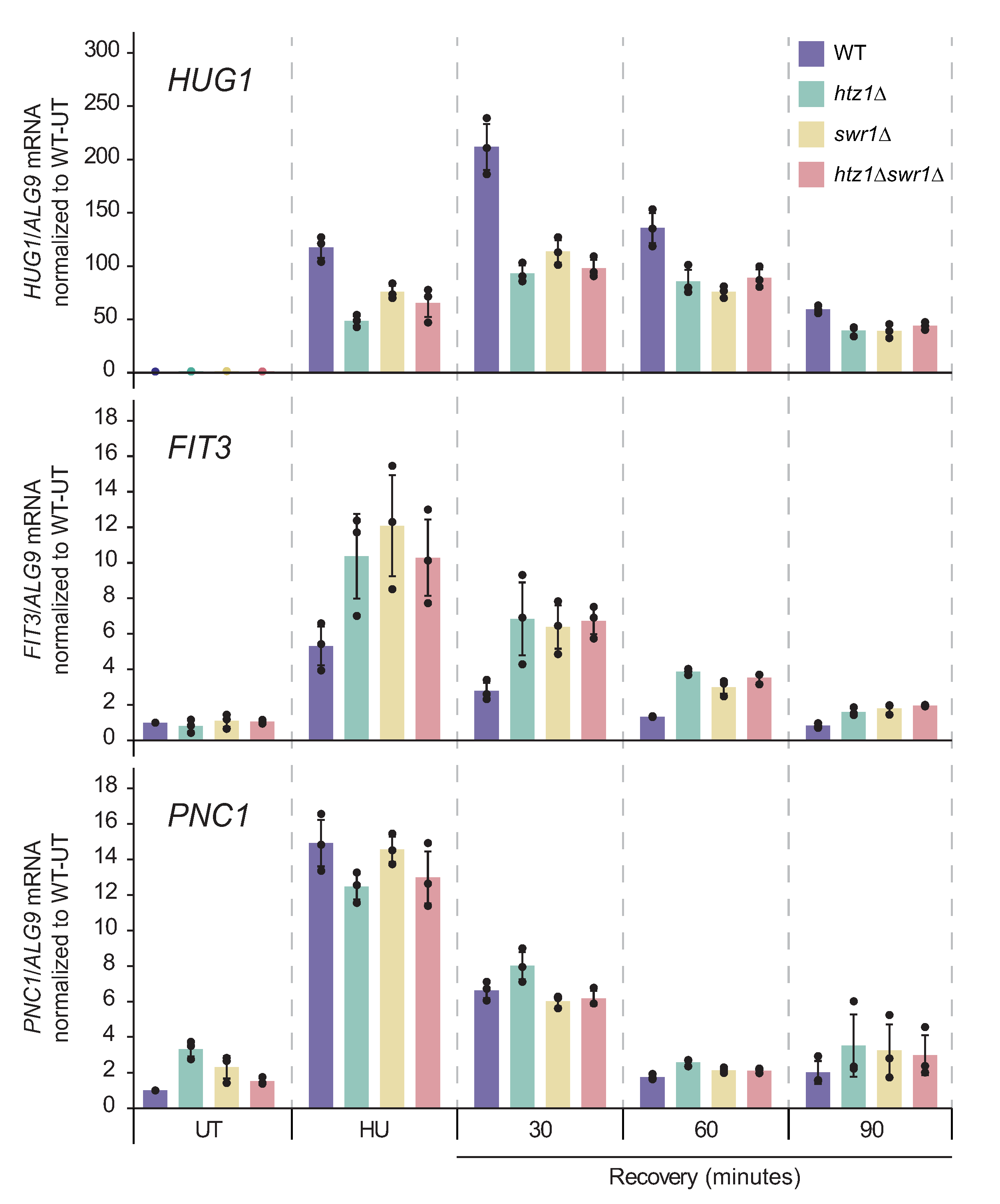

Supplement: S9 Fig — RT-qPCR analysis of HUG1, FIT3, and PNC1 mRNA levels from three replicates were normalized to both ALG9 mRNA levels and to the median wild-type untreated mRNA levels. RNA was extracted from untreated cells (UT), HU-treated cells (HU), and from cells 30, 60, and 90 minutes after HU-removal. (TIF) [file pgen.1011566.s009.tif]

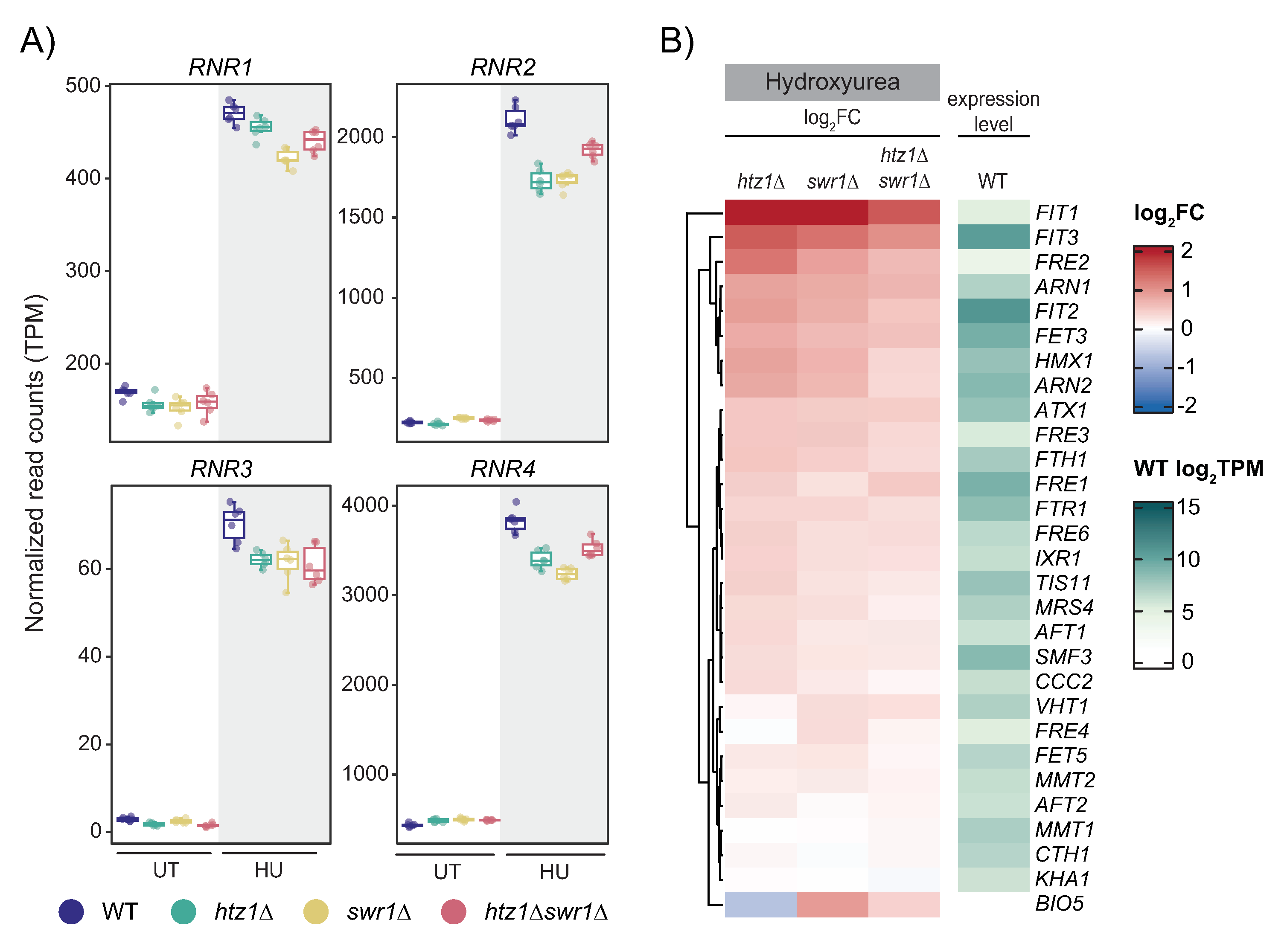

Supplement: S10 Fig — (A) RNR1-4 expression levels in the untreated and HU-treated condition for all four genotypes. (B) Heatmaps illustrate the log2FC of gene expression between the mutants and wild-type in the HU-treated condition for 29 genes in the iron regulon. The average expression level of each gene in wild-type under untreated conditions is presented on the right. (TIF) [file pgen.1011566.s010.tif]

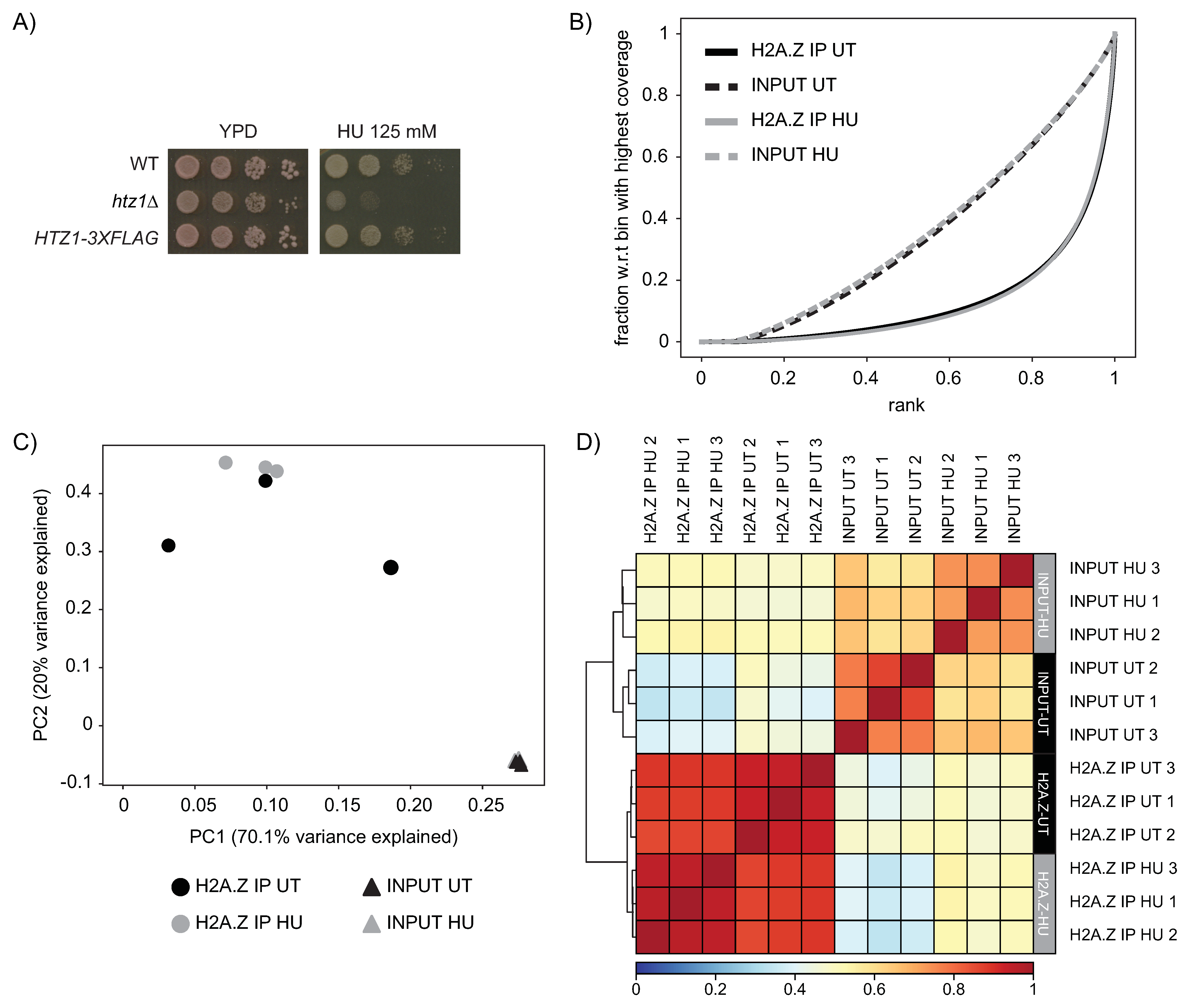

Supplement: S11 Fig — (A) The addition of a 3X-FLAG tag to H2A.Z did not affect cellular growth in HU. Cells were 10-fold serially diluted, spotted onto YPD media with 125 mM HU. (B) Fingerprint plots showing that H2A.Z signal in the IP samples could be successfully differentiated from background signal (INPUT samples). (B) Biplot of PC1 and PC2 generated from a Principal Component Analysis (PCA) of aligned reads. Each data point represents a single biological replicate (n = 3). (C) Spearman’s correlation coefficient matrix of aligned reads from H2A.Z IP and INPUT samples show that samples clustered by condition. (TIF) [file pgen.1011566.s011.tif]
